# Multimodality imaging in takotsubo syndrome: a joint consensus document of the European Association of Cardiovascular Imaging (EACVI) and the Japanese Society of Echocardiography (JSE)

**DOI:** 10.1007/s12574-020-00480-y

**Published:** 2020-09-04

**Authors:** Rodolfo Citro, Hiroyuki Okura, Jelena R Ghadri, Chisato Izumi, Patrick Meimoun, Masaki Izumo, Dana Dawson, Shuichiro Kaji, Ingo Eitel, Nobuyuki Kagiyama, Yukari Kobayashi, Christian Templin, Victoria Delgado, Satoshi Nakatani, Bogdan A Popescu

**Affiliations:** 1 Cardiothoracic Vascular Department, University Hospital “San Giovanni di Dio e Ruggi d’Aragona”, Salerno, Italy; 2 Department of Cardiology, Gifu University Graduate School of Medicine, Gifu, Japan; 3 Department of Cardiology, University Hospital Zurich, Zurich, Switzerland; 4 Department of Cardiovascular Medicine, National Cerebral and Cardiovascular Center, Suita, Osaka, Japan; 5 Department of Cardiology and Intensive Care, Centre Hospitalier de Compiegne, Compiegne, France; 6 Division of Cardiology, Department of Internal Medicine, St. Marianna University School of Medicine, Kawasaki, Japan; 7 Department of Cardiology, Aberdeen Cardiovascular and Diabetes Centre, Aberdeen Royal Infirmary and University of Aberdeen, Aberdeen, Scotland, UK; 8 Department of Cardiovascular Medicine, Kobe City Medical Center General Hospital, Kobe, Japan; 9 Department of Cardiology, University Heart Center Lübeck, Medical Clinic II (Cardiology/Angiology/Intensive Care Medicine), Lübeck, Germany; 10 Department of Cardiology, German Center for Cardiovascular Research (DZHK), partner site Hamburg/Kiel/Lübeck, Lübeck, Germany; 11 Department of Digital Health and Telemedicine R&D, Juntendo University and Department of Cardiovascular Medicine, Juntendo University School of Medicine, 2-1-1 Hongo, Bunkyo City, Tokyo, 113-8421 Japan; 12 Department of Cardiovascular Institute, Stanford University, Stanford, CA USA; 13 Department of Cardiology, Leiden University Medical Center, Leiden, The Netherlands; 14 Saiseikai Senri Hospital, Suita, Osaka, Japan; 15 Department of Cardiology, University of Medicine and Pharmacy “Carol Davila,” Euroecolab, Bucharest, Romania; 16 Department of Cardiology, Emergency Institute for Cardiovascular Diseases “Prof. Dr. C. C. Iliescu”, Bucharest, Romania

**Keywords:** Takotsubo syndrome, Stress cardiomyopathy, Echocardiography, Cardiac magnetic resonance, Multimodality imaging

## Abstract

Takotsubo syndrome (TTS) is a complex and still poorly recognized heart disease with a wide spectrum of possible clinical presentations. Despite its reversibility, it is associated with serious adverse in-hospital events and high complication rates during follow-up. Multimodality imaging is helpful for establishing the diagnosis, guiding therapy, and stratifying prognosis of TTS patients in both the acute and post-acute phase. Echocardiography plays a key role, particularly in the acute care setting, allowing for the assessment of left ventricular (LV) systolic and diastolic function and the identification of the typical apical-midventricular ballooning pattern, as well as the circumferential pattern of wall motion abnormalities. It is also useful in the early detection of complications (i.e. LV outflow tract obstruction, mitral regurgitation, right ventricular involvement, LV thrombi, and pericardial effusion) and monitoring of systolic function recovery. Left ventriculography allows the evaluation of LV function and morphology, identifying the typical TTS patterns when echocardiography is not available or wall motion abnormalities cannot be properly assessed with ultrasound. Cardiac magnetic resonance provides a more comprehensive depiction of cardiac morphology and function and tissue characterization and offers additional value to other imaging modalities for differential diagnosis (myocardial infarction and myocarditis). Coronary computed tomography angiography has a substantial role in the diagnostic workup of patients with acute chest pain and a doubtful TTS diagnosis to rule out other medical conditions. It can be considered as a non-invasive appropriate alternative to coronary angiography in several clinical scenarios. Although the role of nuclear imaging in TTS has not yet been well established, the combination of perfusion and metabolic imaging may provide useful information on myocardial function in both the acute and post-acute phase.

**Electronic supplementary material:**

The online version of this article (10.1007/s12574-020-00480-y) contains supplementary material, which is available to authorized users.

## Introduction

Takotsubo syndrome (TTS), also known as takotsubo cardiomyopathy, stress-induced cardiomyopathy, or apical ballooning syndrome, is an acute and transient heart failure syndrome originally reported by Dr Sato in 1991 in a Japanese textbook and by Pavin et al. in Europe in 1997 [1, 2]. Chest pain and/or dyspnoea are the most common symptoms at presentation, whereas diaphoresis and syncope are less frequently observed [3]. TTS generally occurs in post-menopausal women, though in several registries and case series up to 10% of TTS patients are male. Although stressor events (emotional or physical) usually trigger the clinical onset of TTS, in about one-third of cases TTS has also been described without a preceding trigger.

Recently, a classification of TTS according to the type of triggering event has been proposed (Table 1) [4].

**Table 1:** InterTAK classification of takotsubo syndrome based on the type of triggering event (from Ghadri et al. [4])

Class I: takotsubo syndrome related to emotional stress	
Class II: takotsubo syndrome related to physical stress	
IIa: takotsubo syndrome secondary to physical activities, medical conditions, or procedures	
IIb: takotsubo syndrome secondary to neurologic disorders	
Class III: takotsubo syndrome without an identifiable triggering event	

Electrocardiographic abnormalities (ST-T elevation in the majority of cases) resembling those detectable in acute coronary syndromes are common, as well as increased biomarkers reflecting myocyte damage despite unobstructed coronary arteries. For this reason, TTS has been classified as ‘myocardial injury’ in the last universal definition of myocardial infarction and categorized as myocardial infarction with non-obstructive coronary arteries (MINOCA) [4]. The pathophysiology of TTS is still unclear. Neuroendocrine, metabolic, genetic, and inflammatory factors via increased adrenergic stimulation and high level of catecholamine release seem to be involved in the genesis of the reversible myocardial stunning associated with this fascinating syndrome [3]. Initially, multivessel coronary spasm was suspected as a possible cause of this unique wall motion defect, typically characterized by apical ballooning. Iga et al. described the first echocardiographic findings in eight cases with transient left ventricular (LV) segmental asynergy and suggested asynergy was unrelated to coronary artery disease [6]. Now, it is well recognized that a ‘takotsubo-like’ appearance represents only a part of this syndrome and many variant forms have been reported [7–9]. Despite its transient nature, the acute phase of TTS is characterized by a substantial incidence of adverse events such as acute heart failure, cardiogenic shock, and arrhythmias, and considerable in-hospital death (4–5%) [10, 11]. Even at long term, TTS recurrence, cardiac and non-cardiac disorders, and increased mortality have been reported.

Owing to the wide spectrum of clinical presentations, diagnosis of TTS is often challenging. However, early recognition of this syndrome is key to adopting an appropriate therapy. Multimodality imaging is helpful in reinforcing the clinical suspicion of TTS in the early phase, allowing confirmation of the diagnosis, even retrospectively, after ruling out other clinical entities that should be considered in the differential diagnosis. The aim of this consensus document is to discuss and review the utility of multimodality imaging, including left ventriculography, echocardiography, computed tomography (CT), magnetic resonance imaging, and nuclear imaging, in the diagnostic workup of TTS.

## Diagnostic criteria

Several diagnostic criteria for TTS have been proposed, including those issued by the Mayo Clinic, the Japanese guidelines [12], the Tako-tsubo Italian Network, the Gothenburg group, and the Heart Failure Association (HFA) TTS Taskforce of the European Society of Cardiology (ESC). Recently, the InterTAK diagnostic criteria have also been developed [3] that incorporate several different aspects: (i) right ventricular (RV) involvement and other atypical wall motion abnormalities (WMAs); (ii) emotional or physical stress are no longer mandatory features; (iii) neurological disorders and pheochromocytoma are considered as potential triggers for TTS; and finally (iv) the possibility of coexisting significant coronary artery disease and TTS has been confirmed (Table 2). According to the 4th universal criteria of myocardial infarction, the diagnosis of TTS should be based on the absence of coronary artery disease, or, if present (about 15% of cases), it should not be sufficient to explain the observed pattern of regional WMAs. Additionally, criteria for diagnosis of types 1, 2, and 4 myocardial infarction should be excluded [5].

**Table 2:** InterTAK diagnostic criteria for takotsubo syndrome (from Ghadri et al. [3])

1. Patients show transient^a^ left ventricular dysfunction (hypokinesia, akinesia, or dyskinesia) presenting as apical ballooning or mid-ventricular, basal, or focal wall motion abnormalities. Right ventricular involvement can be present. Besides these regional wall motion patterns, transitions between all types can exist. The regional wall motion abnormality usually extends beyond a single epicardial vascular distribution; however, rare cases can exist where the regional wall motion abnormality is present in the subtended myocardial territory of a single coronary artery (focal TTS)^b^	
2. An emotional, physical, or combined trigger can precede the takotsubo syndrome event, but this is not obligatory	
3. Neurologic disorders (e.g. subarachnoid haemorrhage, stroke/transient ischaemic attack, or seizures) as well as pheochromocytoma may serve as triggers for takotsubo syndrome	
4. New ECG abnormalities are present (ST-segment elevation, ST-segment depression, T-wave inversion, and QTc prolongation); however, rare cases exist without any ECG changes	
5. Levels of cardiac biomarkers (troponin and creatine kinase) are moderately elevated in most cases; significant elevation of brain natriuretic peptide is common	
6. Significant coronary artery disease is not a contradiction in takotsubo syndrome	
7. Patients have no evidence of infectious myocarditis^b^	
8. Post-menopausal women are predominantly affected	

^a^Wall motion abnormalities may remain for a prolonged period of time or documentation of recovery may not be possible. For example, death before evidence of recovery is captured

^b^Cardiac magnetic resonance imaging is recommended to exclude infectious myocarditis and to confirm diagnosis of takotsubo syndrome

## Clinical course

Although initial studies suggested that prognosis of TTS is benign (in-hospital death 1–1.7%) [13, 14], recent data have shown higher in-hospital mortality rates (3.5–5%) [9, 15, 16]. Clinical characteristics that were associated with in-hospital adverse events or death included male gender, the presence of physical triggers, acute neurologic or psychiatric disease, the first troponin level, and a LV ejection fraction (EF) of <45% [7]. Multiple unfavourable echocardiographic findings (described in the next sections) were associated with in-hospital complications, with evidence of RV involvement being associated with poor long-term outcomes [15].

## Cardiac catheterization: coronary angiography and left ventriculography

Coronary arteries are generally normal or near-normal in TTS. Although echocardiography is the first-line imaging modality in patients with suspicion of TTS, assessment of coronary anatomy is crucial in TTS diagnostic workup to rule out alternative diagnoses. As recommended by current guidelines on acute coronary syndromes (ACS), cardiac catheterization should be performed in all patients with acute cardiac ischaemia and ST-segment elevation or in patients without ST-segment elevation according to the individual risk profile, especially for patients with a low-intermediate probability of TTS [10, 17–21].

**Figure 1:**
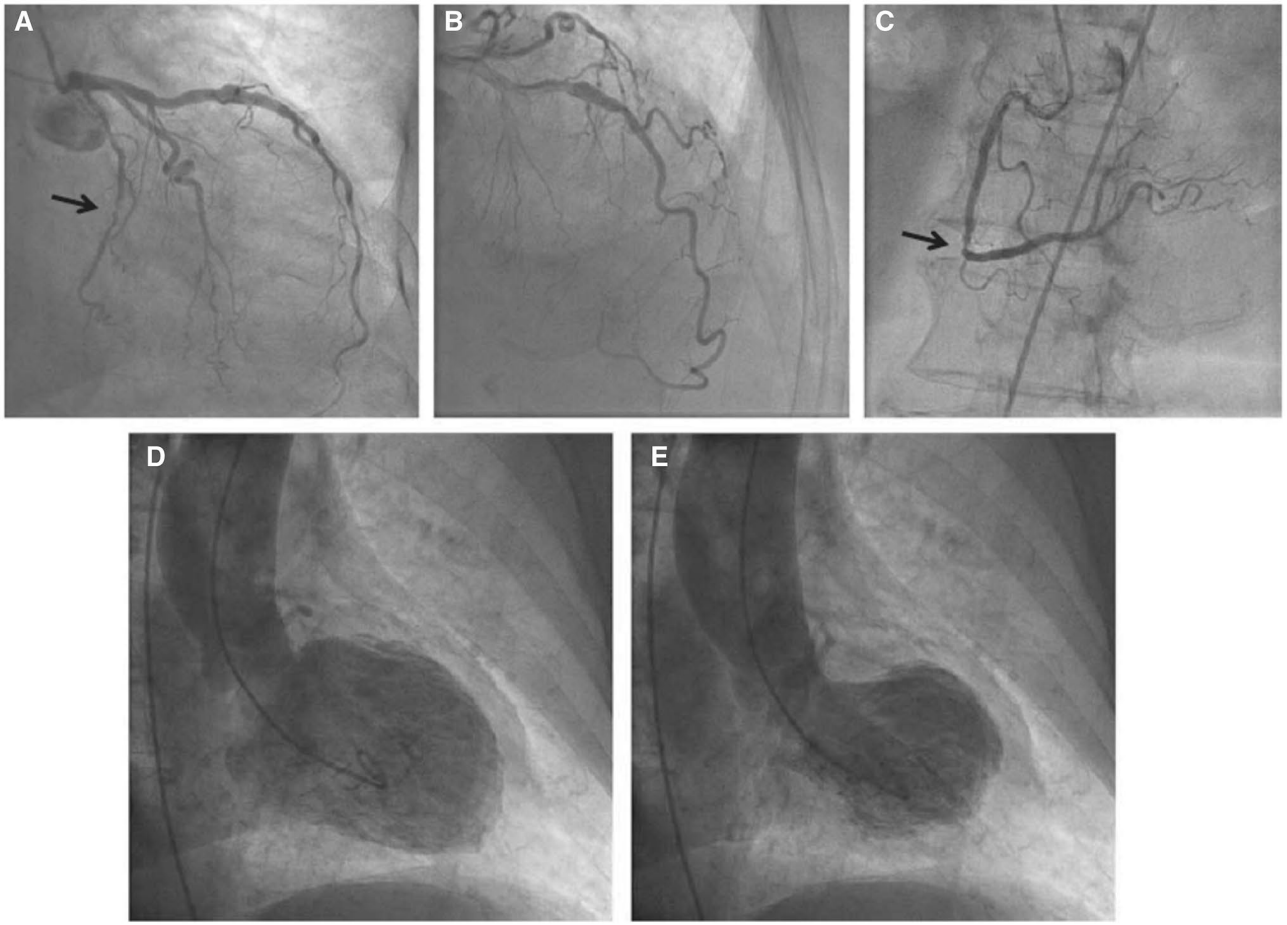
Patient with takotsubo syndrome (TTS) and concomitant significant coronary artery disease. Caudal (**a**) and cranial (**b**) angiographic views of the left coronary artery. Left cranial view (**c**) of the right coronary artery. Note the significant stenosis in the circumflex artery (arrow, **a**) and in the right coronary artery (arrow, **c**). Typical apical ballooning pattern of TTS at left ventriculography (**d** and **e**). (Reprinted with permission from Parodi et al. [18])

Coronary anatomy should be carefully evaluated using multiple angiographic views, ensuring that all lesions have been assessed in at least two orthogonal projections. Obstructive atherosclerotic plaques can be observed in about 1 in 10 of TTS patients [3, 18]. It must be emphasized that obstructive single-vessel coronary lesions are not an absolute exclusion criterion for diagnosis, since LV WMAs usually extend beyond a single epicardial vascular distribution in TTS, whilst equally non-obstructive plaques can cause myocardial infarction (Fig. 1) [10, 18, 19].

**Figure 2:**
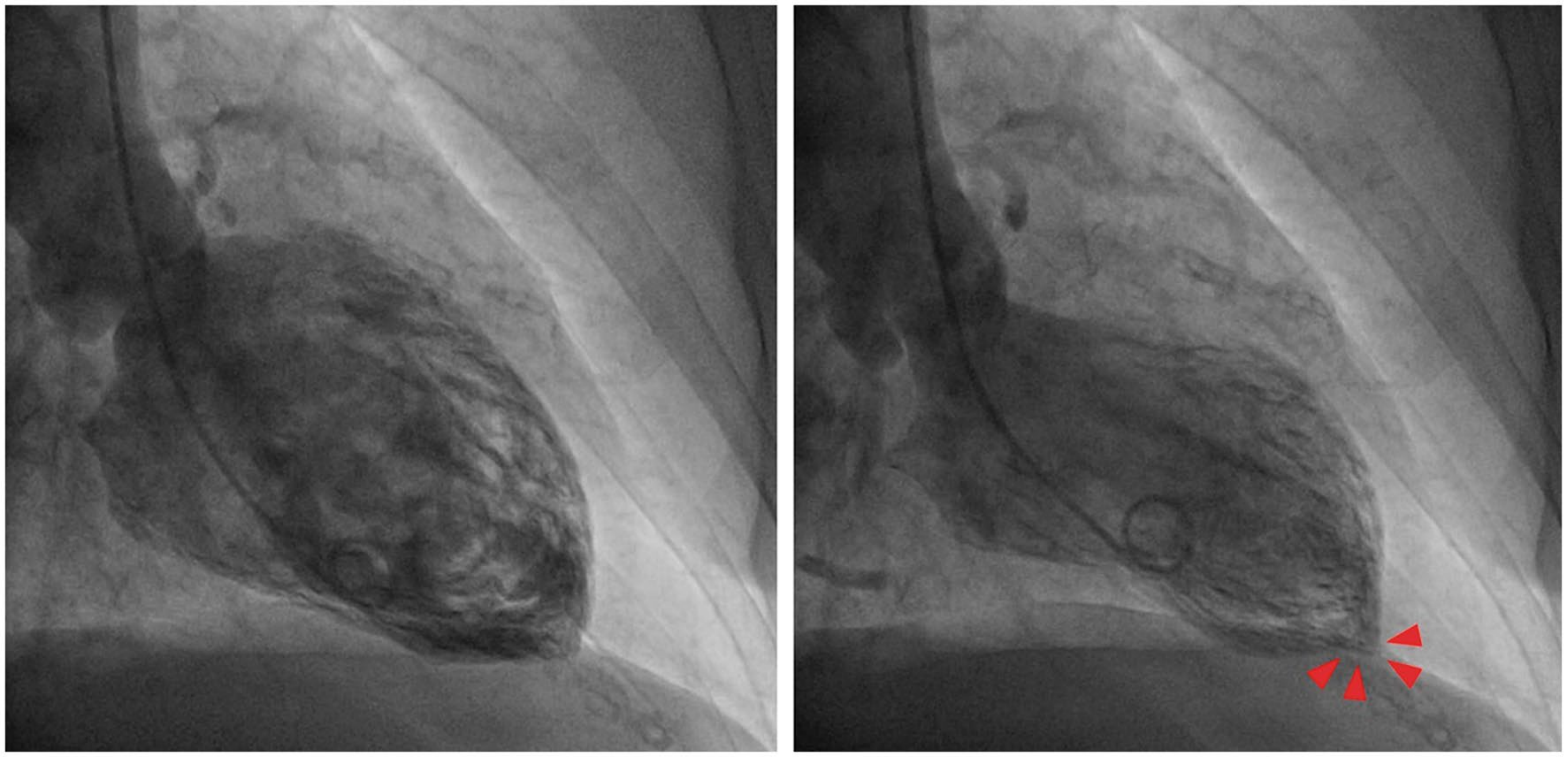
Left ventriculography of a patient with takotsubo syndrome and typical apical ballooning pattern. Note the presence of the ‘apical nipple’ sign, a small area just at the apex with preserved contractility (arrow heads)

**Figure 3:**
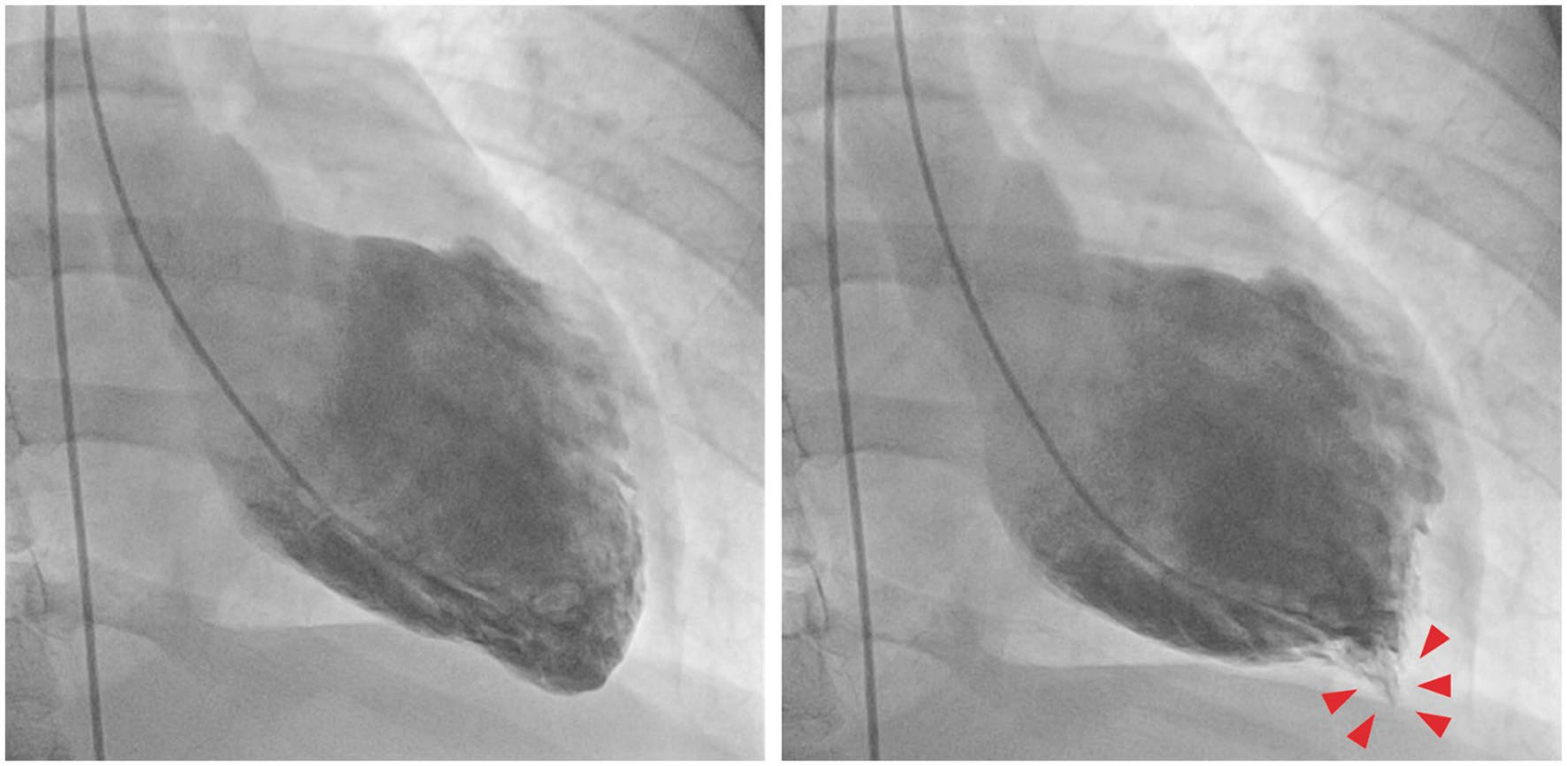
Left ventriculography of a patient with takotsubo syndrome and midventricular ballooning pattern. Note the left ventricular apex in end-systole resembling hawk’s beak appearance (arrow heads)

**Figure 4:**
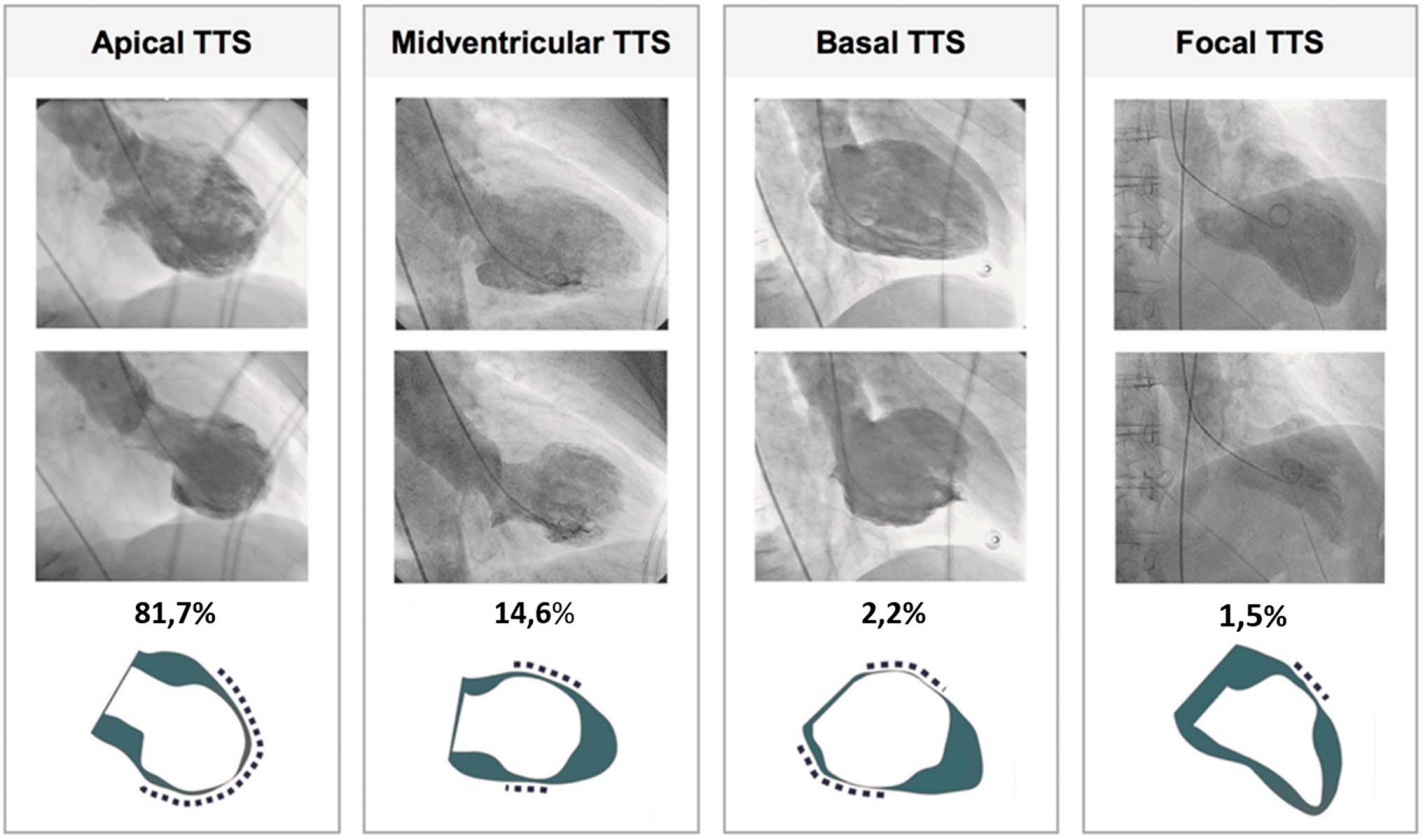
Left ventriculography in the right anterior oblique projection demonstrates four different morphological patterns of takotsubo syndrome (TTS): apical, midventricular, basal, and focal type. (Modified and reprinted with permission from Templin et al. [7])

The diagnosis of TTS can also be established during cardiac catheterization through LV opacification. Biplane left ventriculography in the right and left anterior oblique projections allows for the assessment of nine LV segments and differentiation of TTS from anterior ST-elevation myocardial infarction (STEMI) in the majority of cases [19, 22]. The ‘apical nipple’ sign, a very small zone with preserved contractility of the LV apex, has been described on left ventriculography in about 30% of patients with TTS and typical apical ballooning (Fig. 2) [23]. This sign can be a useful additional tool to discriminate TTS from acute anterior STEMI, in which the phenomenon is not observed. Similarly, in patients with the mid-ventricular variant, systolic contraction of the apex can configure the hawk’s beak appearance by ventriculography (Fig. 3) [24, 25]. In addition, the LV chamber silhouette depicted by the right anterior oblique view may show the typical apical ballooning or one of the variant morphologic patterns, which are suggestive of TTS diagnosis (Fig. 4) [7, 26]. Given the transient and reversible course of WMAs, left ventriculography should be performed in all patients with suspected TTS, especially if no transthoracic echocardiogram is available or in patients with poor acoustic window [8]. LV opacification may also be useful to identify possible mechanical complications such as acute mitral regurgitation (MR) or LV apical thrombi, which have prognostic implications [14]. Before removal, the catheter should be withdrawn slowly from the LV cavity in order to assess invasively intraventricular pressure gradients and possibly detect LV outflow tract obstruction (LVOTO), particularly in patients with haemodynamic instability [26].

Although not specific for TTS, coronary anatomical variants, including myocardial bridging or long and tortuous arteries, are common findings on CA [27]. Likewise, a high thrombolysis in myocardial infarction frame count is frequently observed and has been associated with coronary microcirculation dysfunction with consequent slow coronary flow [28, 29]. Beyond the identification of significant coronary lesions using conventional angiography, TTS diagnosis requires the exclusion of any other epicardial mechanisms potentially involved in other types of MINOCA such as plaque rupture or erosion and above all spontaneous coronary artery dissection [30]. The use of intravascular imaging modalities, e.g., optical coherence tomography and intravascular ultrasound, may help to clarify alternative aetiologies of myocardial injury [31, 32]. However, given the technical complexity and high costs, the application of these tools should be considered only in selected cases with unclear or inconclusive angiographic findings.

Key points

- CA should be performed in ACS according to guidelines in all STEMI and intermediate to very high-risk non-STEMI presentations.
- Obstructive coronary stenosis does not represent an absolute exclusion criteria for TTS, but if coronary artery disease is present, it should not be sufficient to explain the observed regional WMAs.
- Biplane left ventriculography allows assessment of LV function and morphology in TTS.
- In selected cases, intravascular imaging and functional testing to rule out vasospasm may be helpful for the differential diagnosis.

## Role of standard echocardiography

Transthoracic echocardiography (TTE) is the first-line imaging modality for the evaluation of patients with TTS [33]. If suspicion of TTS arises, echocardiography should be promptly performed because it provides useful information about systolic and diastolic function, RV involvement, haemodynamic status, mechanical complications, pulmonary artery systolic pressure, pericardial effusion, and LV intraventricular thrombi [26]. Echocardiographic findings are also helpful to identify patients at higher risk of adverse outcome and to monitor regression of WMAs (Tables 3 and 4).

**Table 3:** Main echocardiographic findings in takotsubo syndrome

LV systolic function	Marked reduction in LVEF on admission with improvement at short term
LV WMAs	•Independent of the distribution of epicardial coronary artery (circumferential pattern)
	•Apical ballooning
	•Variant form: mid-ventricular ballooning; inverted TTS
RV involvement	Reverse McConnell sign (biventricular ballooning)
Speckle-tracking	Circumferential impairment of LV longitudinal and radial strain
Coronary flow	•Preserved distally to the coronary artery
	•Coronary flow reserve is impaired in the acute phase

EF, ejection fraction; LV, left ventricular; RV, right ventricular; TTS, takotsubo syndrome; WMAs, wall motion abnormalities.

**Table 4:** Echocardiographic findings of high risk in takotsubo syndrome

Low CO	
LVEF <35%	
Elevated *E*/*e*′ ratio	
LVOTO	
MR >2+/4+	
RV involvement	
LV thrombi	
Pericardial effusion	
Left ventricular wall rupture	

CO, cardiac output; EF, ejection fraction; LV, left ventricular; LVOTO, left ventricular outflow tract obstruction; MR, mitral regurgitation; RV, right ventricular.

### Wall motion abnormalities and morphologic anatomical variants

Firstly, TTE may help to detect the distribution of WMAs and determine the morphologic anatomical variant [8, 34].

In the ‘apical ballooning’ form, the apex and/or mid-ventricular myocardial segments are diffusely akinetic while basal segments are hyperkinetic (Supplementary data online, *Videos S1* and *S2*) [8, 33–35]. Conversely, in the *‘*midventricular’ form, akinesis is confined to the mid segments whereas the apical segments are normal or only mildly hypokinetic [8]. These two morphologic variants account for the vast majority of TTS and are both characterized by myocardial dysfunction involving the opposite LV wall beyond the territory of a single coronary artery distribution [36]. This *circumferential pattern* of myocardial dysfunction represents a hallmark of TTS diagnosis [33] and should always be described in the echo report.

Other rare forms (i.e. basal or focal) are difficult to diagnose with certainty on echocardiography alone; in particular, the focal type is usually suspected only after exclusion of other possible aetiologies in the differential diagnosis.

RV involvement should be assessed in parasternal long-axis, apical and subcostal four-chamber views, adjusting the echo transducer to the level of the RV chamber to achieve optimal visualization of its size and endocardial borders (Supplementary data online, *Videos S3*). RV involvement [37] is usually identified by the detection of severe akinesis or dyskinesis, localized exclusively at the apical and/or mid RV segments (biventricular ballooning) [38], with sparing of the basal segments (‘reverse McConnell’s sign’) [39].

Biventricular ballooning reinforces the diagnostic suspicion of TTS, especially if pulmonary hypertension and acute pulmonary embolism are excluded. RV involvement has been associated with adverse in-hospital outcomes [14, 40]. It should be taken into account that in some cases of anterior myocardial infarction with wrap-around morphology of the left anterior descending artery (LAD), RV involvement beside LV apical akinesia can be appreciated [41].

### Systolic and diastolic function

Systolic function should be carefully defined since a marked reduction in LVEF on admission is associated with adverse in-hospital outcomes, particularly in elderly patients and in those with physical stressors [42, 43]. Recent data also suggest that advanced systolic dysfunction could negatively affect outcomes even after hospital discharge [44].

Spontaneous myocardial function recovery, typically associated with TTS, should ideally be monitored with daily echocardiography during the acute phase [45].

Additionally, echocardiographic diastolic indices, namely the *E*/*e*′ ratio, should be assessed early and systematically to identify patients at higher risk for acute heart failure and to guide appropriate management. Given that diastolic dysfunction is also transient and reversible, improvements in the *E*/*e*′ ratio can be used as an additional marker of LV functional recovery [8, 14].

### Mechanical complications (left ventricular outflow tract obstruction and reversible mitral regurgitation)

Echocardiography in the acute phase allows the prompt identification or exclusion of several important mechanical complications, including LVOTO, MR, and myocardial wall rupture [46–48].

**Figure 5:**
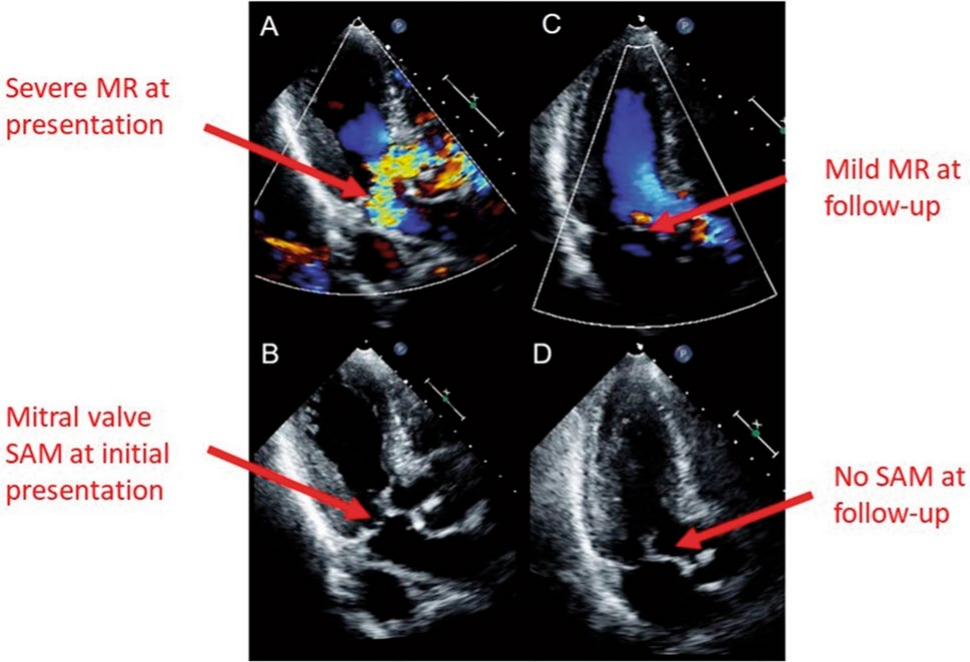
Two-dimensional transthoracic echocardiography, apical long-axis views, in patients with mitral regurgitation (MR) due to systolic anterior motion (SAM) of the mitral valve. (*A*) Severe MR at initial presentation. (*B*) SAM of the mitral valve at initial presentation. (*C*) Only mild MR was found at follow-up. (*D*) SAM of the mitral valve was not found at follow-up. (Modified from Izumo et al. [55])

Dynamic LVOTO, defined as an intraventricular gradient >25 mmHg, results from basal hypercontractility in the small LV cavity with asymmetric hypertrophy of the interventricular septum [49]. Its prevalence ranges from 12.8% to 25% [50]. The degree of LVOTO is variable and reversible depending on loading conditions and myocardial function recovery. It may be associated with systolic anterior motion of the mitral valve (SAM), leading in turn to MR (Supplementary data online, *Videos S4–S6*; Fig. 5) [51, 52]. Since concomitant LVOTO and MR further contribute to haemodynamic instability, early echocardiographic identification has important therapeutic implications. In this clinical scenario, administration of inotropic agents and diuretics results in enhanced basal contractility and volume depletion, respectively. These both increase intraventricular pressure gradients with worsening of the haemodynamic status, ultimately leading to acute heart failure and cardiogenic shock [53]. It is therefore recommended to discontinue inotropic agents and administer fluids while monitoring the patient’s haemodynamic status. If TTE confirms the persistence of SAM with low cardiac output, current recommendations suggest the use of LV assist devices to improve cardiac output [3, 10, 17, 54].

**Figure 6:**
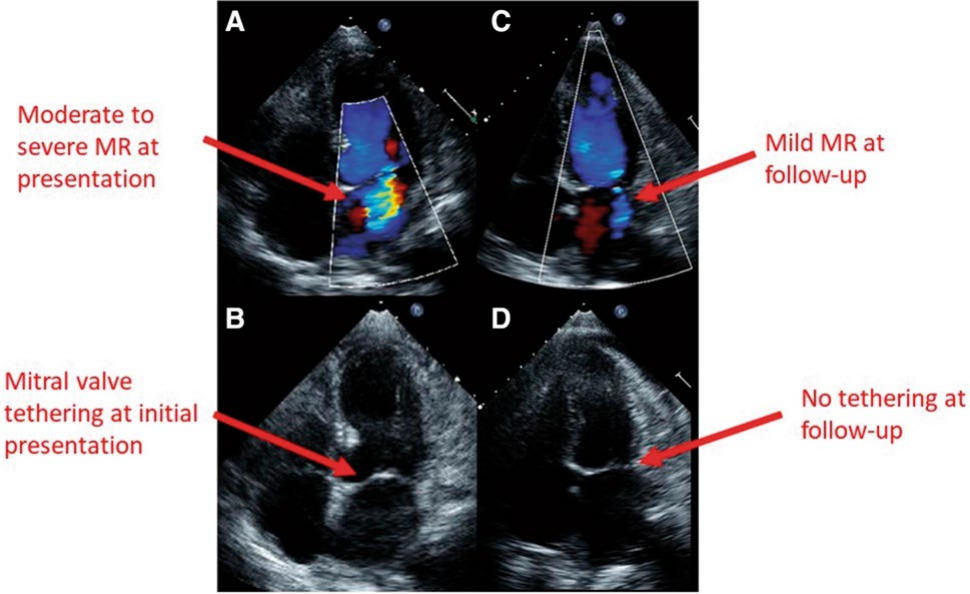
Two-dimensional transthoracic echocardiography, apical four-chamber views, in patients with functional mitral regurgitation (MR). (*A*) Moderate to severe MR at initial presentation. (*B*) Mitral valve tethering at initial presentation. (*C*) Only mild MR was found at follow-up. (*D*) Mitral valve tethering was not found at follow-up. (Modified from Izumo et al. [55])

Beyond SAM related to LVOTO, another possible cause of transient and reversible MR is leaflet tethering secondary to papillary muscle displacement in a dilated left ventricle with severely impaired LV systolic function (Fig. 6) [55, 56]. Transient moderate to severe MR has been reported in about 20–25% of TTS patients and is associated with advanced Killip class [14, 51]. Also, pulmonary artery systolic pressure should be assessed by TTE at every follow-up (FU) exam, particularly in TTS patients with signs or symptoms of heart failure.

### Intraventricular thrombi

Extensive apical myocardial dysfunction and reduced intraventricular systolic flow velocity are predisposing factors for thrombus formation. In patients with TTS, mural or pedunculated thrombi visualized at the apex during the first 2 days have an incidence of 1–2% and may cause stroke or systemic embolization (renal or lower limb embolism) in approximately one-third of cases [57]. After thrombus detection, therapy with heparin followed by oral anticoagulation should be instituted, and serial TTE should be performed until thrombus resolution and myocardial contractility recovery [8]. Use of real-time 3D echocardiography or contrast agents may also be helpful in detecting small thrombi [8, 58].

### Left ventricular wall rupture

LV free wall or interventricular septum rupture is a very rare (<1%) life-threatening complication burdened by high mortality rates due to cardiogenic shock and/or cardiac tamponade. In the presence of clear haemodynamic instability, prompt echocardiographic detection of this complication is important to favour rapid referral for surgery [59, 60].

### Pericardial effusion

Pericardial effusion is rarely described in TTS [61, 62] and is more frequently associated with an inflammatory disease of the myocardium and/or pericardium. If pericardial effusion is detected, further investigation by cardiac magnetic resonance (CMR) should be performed to investigate the differential diagnosis of myocarditis [17, 26].

Key points

- Echocardiography is the first-line imaging modality in patients with suspected TTS.
- In diagnostic workup, echocardiography is useful to recognize the apical-midventricular ballooning and circumferential patterns of WMA distribution.
- Echocardiography is useful to monitor the recovery of systolic function and to detect the occurrence of complications (i.e. LVOTO, reversible moderate to severe MR, RV involvement, LV thrombi, and pericardial effusion).
- RV involvement reinforces the diagnostic suspicion of TTS and has been associated with adverse in-hospital outcome.

## Strain imaging by speckle tracking echocardiography

**Figure 7:**
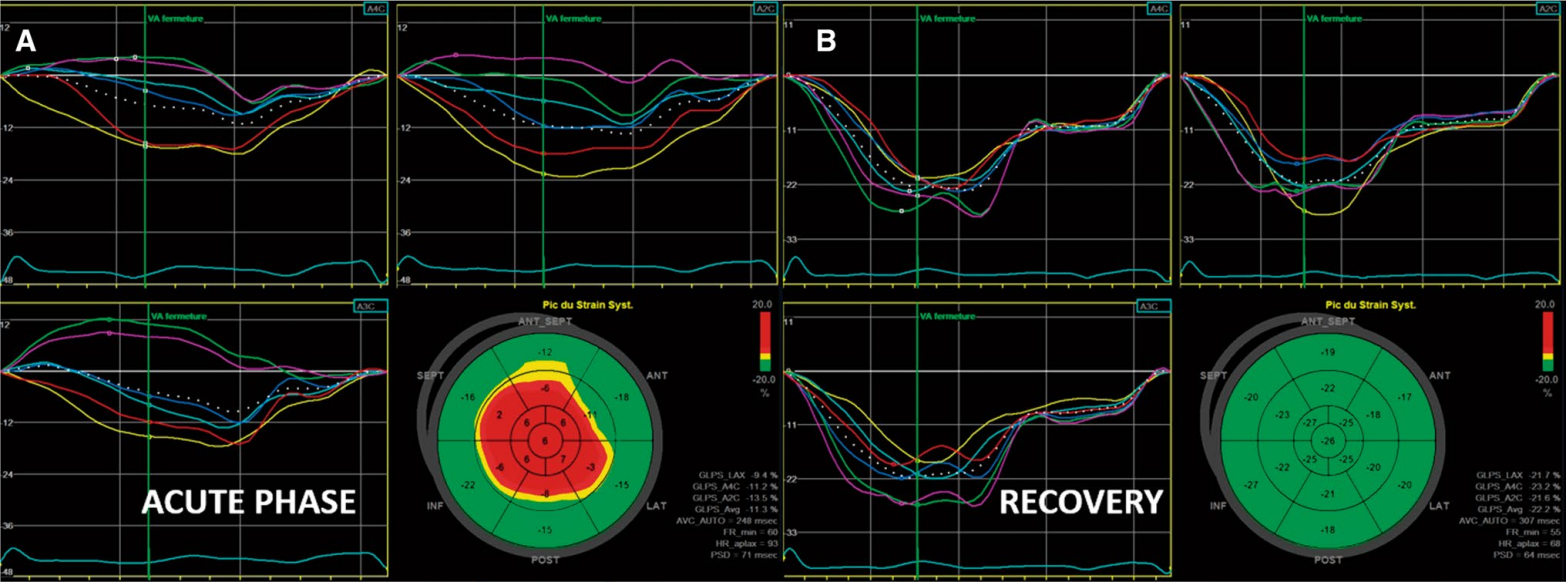
Longitudinal strain in a patient with typical type of takotsubo syndrome demonstrating left ventricular strain acquisition from standard apical view. Apical ballooning and a base to apex circular gradient of strain are appreciated by the bull’s eye plot, which recovers to normal at follow-up

**Figure 8:**
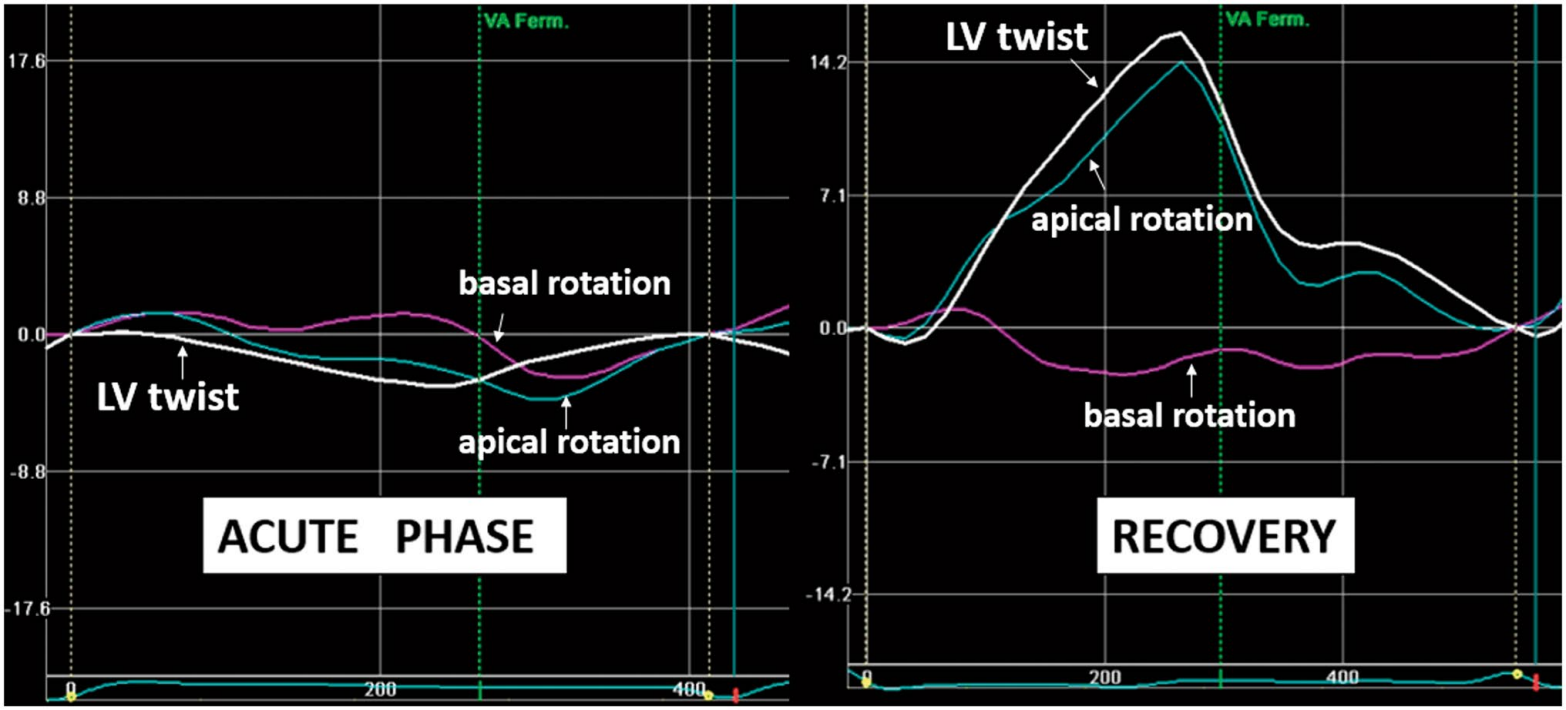
Impaired left ventricular (LV) twist by speckle tracking. In the acute phase, LV twist is significantly impaired mainly due to impaired apical function

Strain imaging by speckle tracking echocardiography allows assessment of global and regional myocardial function in all three layers of myocardium, and which can be applied to assess strain in the longitudinal, circumferential, and radial directions. Owing to the ability of strain imaging to objectively depict the transient impairment of myocardial deformation with circular involvement of the opposite LV walls (circumferential pattern), speckle tracking assessment in TTS patients should be performed, whenever possible, even in the acute phase. Furthermore, it is a useful tool to monitor myocardial functional recovery [63–73] (Fig. 7). LV twist is also transiently impaired in the acute phase of typical TTS, mainly due to apical dysfunction, with secondary alterations in the normal rotational pattern of contraction [74] (Fig. 8). LV early untwisting rate, an index of diastolic function, is also decreased in the acute phase. Recent studies have further demonstrated RV involvement in TTS as an important finding associated with worse outcome [75–80]. Traditional echocardiographic parameters used to assess RV function, such as RV fractional area change and tricuspid annular plane systolic excursion, do not account for contraction abnormalities of the mid- or apical RV segments and are instead predominantly affected by basal hyperkinesis. RV free wall longitudinal strain is therefore recommended in order to detect even small areas of myocardial dysfunction hardly recognizable by visual assessment and/or by other traditional echocardiographic parameters. Conversely, RV global longitudinal strain should not be assessed because involvement of the interventricular septum can be misleading [81].

WMAs and LVEF usually recover to normal within a few weeks. However, several studies have shown that significant mechano-temporal alterations affecting both systole (global longitudinal strain and apical circumferential strain) and diastole (untwist rate and time to peak untwisting), reduced exercise capacity, increased native T1 mapping values and impaired cardiac energetics can persist for months after the acute phase, despite normalization of LVEF and volumes [67, 82].

The long-term prognosis of patients who experienced TTS seems to be worse than that in the general population; the underlying mechanism may be partly due to persistence of oedema/inflammation, with residual functional abnormalities observed on strain imaging [16, 46, 68, 74].

Together with other clinical, functional, and metabolic testing, these studies provide evidence that structural and functional myocardial abnormalities often persist after TTS. These findings do not rule out the diagnosis of TTS and emphasize the need for systematic follow-up. At 1 month from TTS onset, a clinical and echocardiographic follow-up is mandatory: in case of persisting WMAs, ECG changes, elevated levels of B-type natriuretic peptide and/or symptoms, a second follow-up visit should be scheduled at 3 months, conversely the patient should be evaluated at 6 months and 1 year, and then annually. Careful evaluation of persistent systolic and diastolic alterations using speckle tracking echocardiography could add important prognostic information and aid clinical decision-making.

## Transthoracic coronary Doppler

**Figure 9:**
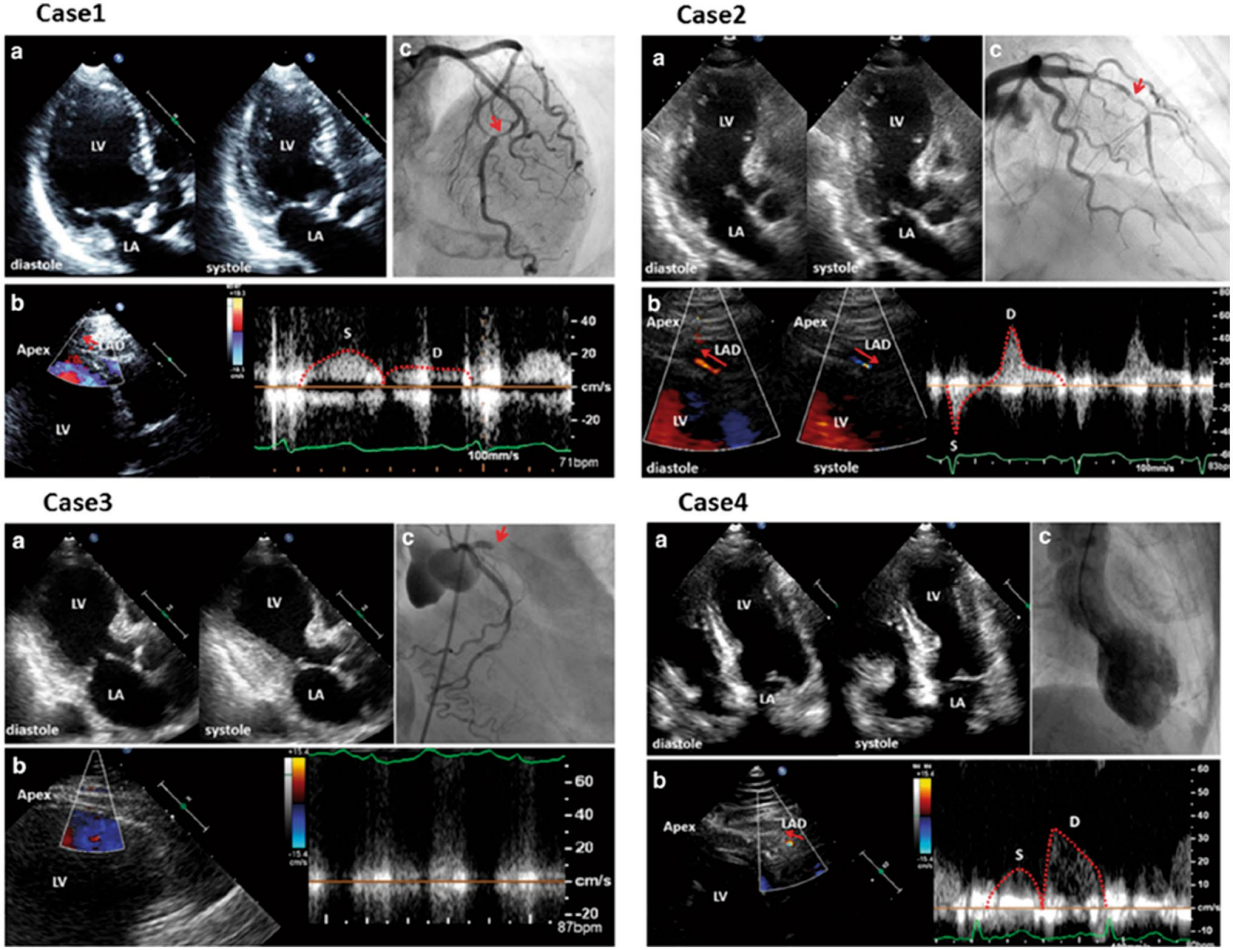
Visualization of coronary flow in acute myocardial infarction (AMI) and takotsubo syndrome (TTS). Cases 1–3 indicate coronary flow pattern in AMI. Systolic-dominant flow pattern with reduced diastolic flow velocity in Case 1, to-and-from pattern in Case 2, and absence of coronary flow in Case 3, all indicate severe stenosis/occlusion of the left anterior descending artery (LAD). In Case 4, antegrade diastolic coronary flow with reduced deceleration time, typical of TTS in the acute phase. LA, left atrium; LV, left ventricle. (Modified and reprinted with permission from Watanabe [84])

Visualization of coronary flow in the distal part of the LAD by transthoracic Doppler can help to differentiate TTS from acute myocardial infarction (AMI) due to LAD occlusion (Fig. 9) [9, 83–85] and also provides non-invasive physiological information on coronary blood flow. Through the ratio of hyperaemic to baseline diastolic peak flow velocity in the distal LAD, this method also allows the assessment of coronary flow reserve after administration of coronary vasodilator agents (e.g. as adenosine or dipyridamole) [86]. In the acute phase of TTS, coronary flow reserve is reduced due to transient coronary microcirculation impairment and recovers prior to LV wall motion normalization [85, 87–90]. The decrease in coronary flow reserve in TTS seems to be milder than that in AMI, although TTS has worse WMAs than AMI, suggesting that microvascular dysfunction is not the only mechanism of TTS [91].

In daily practice, this tool is of limited value, although it may be useful for monitoring the recovery of coronary flow reserve in doubtful cases.

## Contrast echocardiography

**Figure 10:**
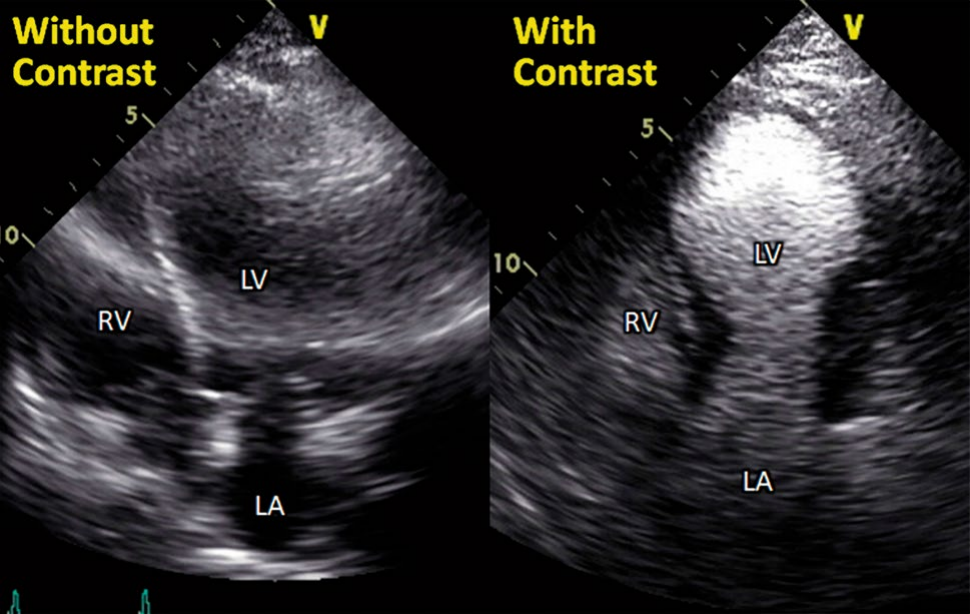
Opacification of wall motion abnormalities by contrast echocardiography. Left and right panels show apical four-chamber views with and without contrast agent in a case of takotsubo syndrome. Contrast echocardiography helps opacification of wall motion abnormalities, especially in patients with poor echo images. LA, left atrium; LV, left ventricle; RV, right ventricle

In clinical practice, the most important application of contrast echocardiography is LV cavity opacification for the detection of WMAs [92] (Fig. 10) and the determination of the morphologic pattern of TTS in patients with poor acoustic window. Furthermore, the use of ultrasound contrast agents improves the detection of intraventricular thrombi, directing prompt introduction of anticoagulant therapy [9]. Finally, contrast echocardiography may be used to improve visualization of coronary flow by TTE with colour and pulsed wave Doppler [83, 85,^86^].

Of note, myocardial contrast echocardiography performed within 24 h after TTS onset revealed reversible perfusion defects in involved segments, which improved faster than WMAs [91].

## 3D echocardiography

**Figure 11:**
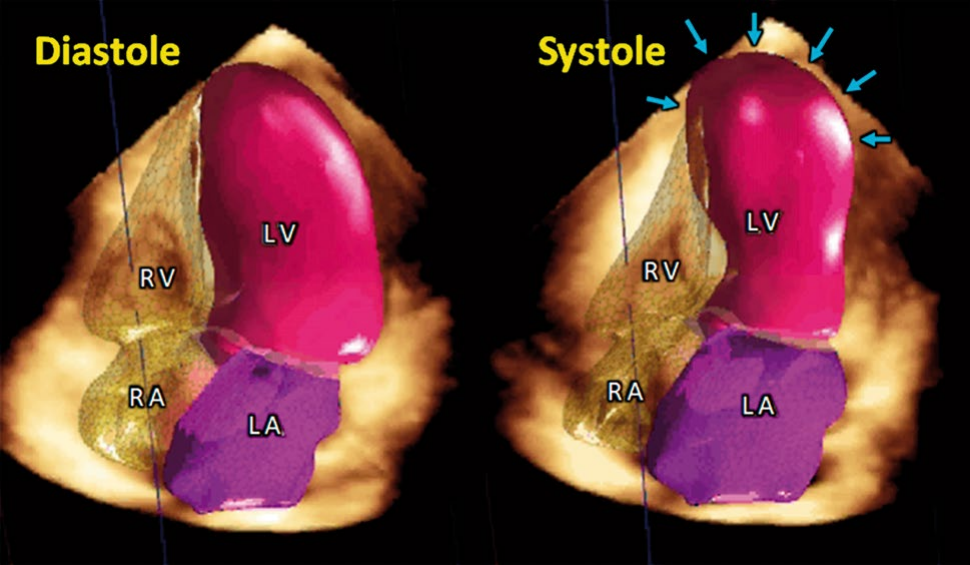
Simultaneous three-dimensional modelling of cardiac chambers. Latest echo machines enable automated 3D quantification of chamber volumes and ejection fractions of the four cardiac chambers. 3D quantification has been reported to have higher accuracy and reproducibility than two-dimensional echocardiography. LA, left atrium; LV, left ventricle; RA, right atrium; RV, right ventricle

**Figure 12:**
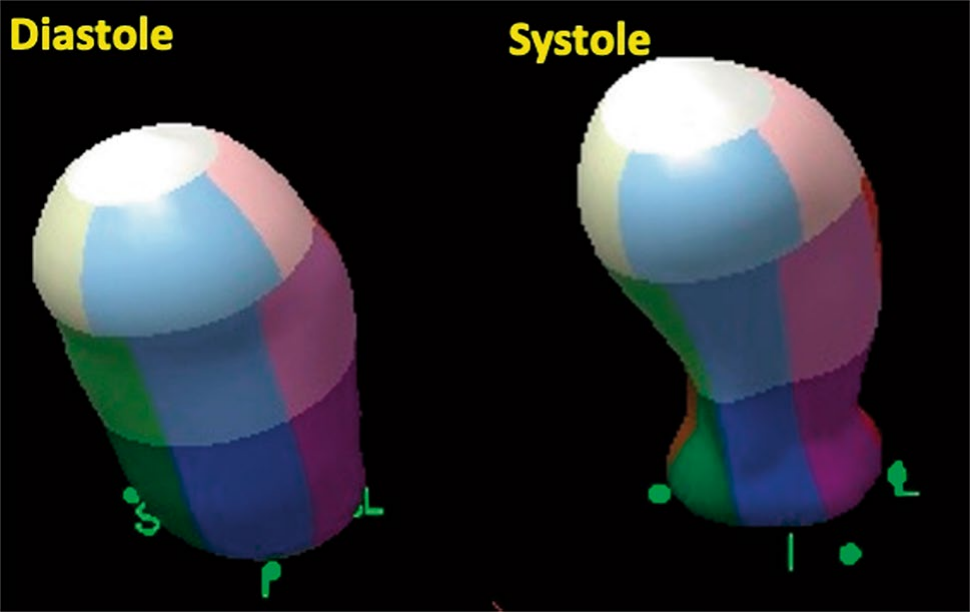
3D assessment of wall motion abnormality. In addition to quantification of volumes, 3D echocardiography is useful for visual confirmation of patterns of wall motion abnormality. The left and right panels indicate an end-diastolic and end-systolic phase, which clearly depicts apical ballooning with basal hyperkinetic motion

3D echocardiography is a useful method to quantify chamber measurements with higher accuracy and reproducibility than 2D echocardiography [93] (Figs. 11 and 12). 3D visualization may enable more objective assessment of regional WMAs and EF, especially in the right ventricle because of its complex morphology [94]. As 2D echocardiography has a limited ability to detect RV involvement [79], 3D echocardiography might help identify RV involvement [95] and improve risk stratification in patients with TTS. However, there is a paucity of data and further studies are warranted.

These ‘advanced’ echocardiography techniques, including strain imaging, coronary flow assessment, contrast echocardiography, and 3D echocardiography, are emerging and hold promise in improving our understanding of the pathophysiology underlying TTS. However, it should be acknowledged that the clinical utility of these techniques has not been fully established and physicians should not make clinical decisions solely on this basis.

Key points

- LV mechanics are profoundly and transiently impaired in TTS, in a pattern that differs from AMI.
- Whilst EF and WMAs resolve in TTS, some subtle wall strain abnormalities can persist.
- The coronary microcirculation is transiently compromised in TTS, but its physiological role is still unclear.
- Contrast echocardiography should be used in patients with poor acoustic window to better define WMAs and the presence of intraventricular thrombus.
- Compared to 2D echocardiography, 3D echocardiography enables more accurate assessment of regional WMAs and may be a useful tool to better detect and assess RV involvement, even in the acute care setting.
- As an additional prognostic tool, RV strain could be useful in assessing the extent of RV involvement.

## Cardiac magnetic resonance

Although echocardiography and left ventriculography have mainly been used to diagnose LV WMAs in patients with TTS, CMR plays an important role in the comprehensive assessment of the functional and structural changes that occur in patients with clinical suspicion of TTS [96]. CMR represents the reference standard for qualitative and quantitative assessment of regional WMAs and accurate quantification of right and LV volumes and function. Other additional abnormalities, including pericardial effusion, pleural effusion, and intraventricular thrombi, can also be easily appreciated. However, the major strength of this imaging technique is myocardial tissue characterization, due to its unique ability to provide different markers of myocardial injury. The detection of oedema/inflammation rather than fibrosis or necrosis is useful to distinguish reversible from irreversible myocardial damage and is helpful in guiding the differential diagnosis between TTS, other MINOCA presentations and ACS.

### Ventricular morphology and function

**Figure 13:**
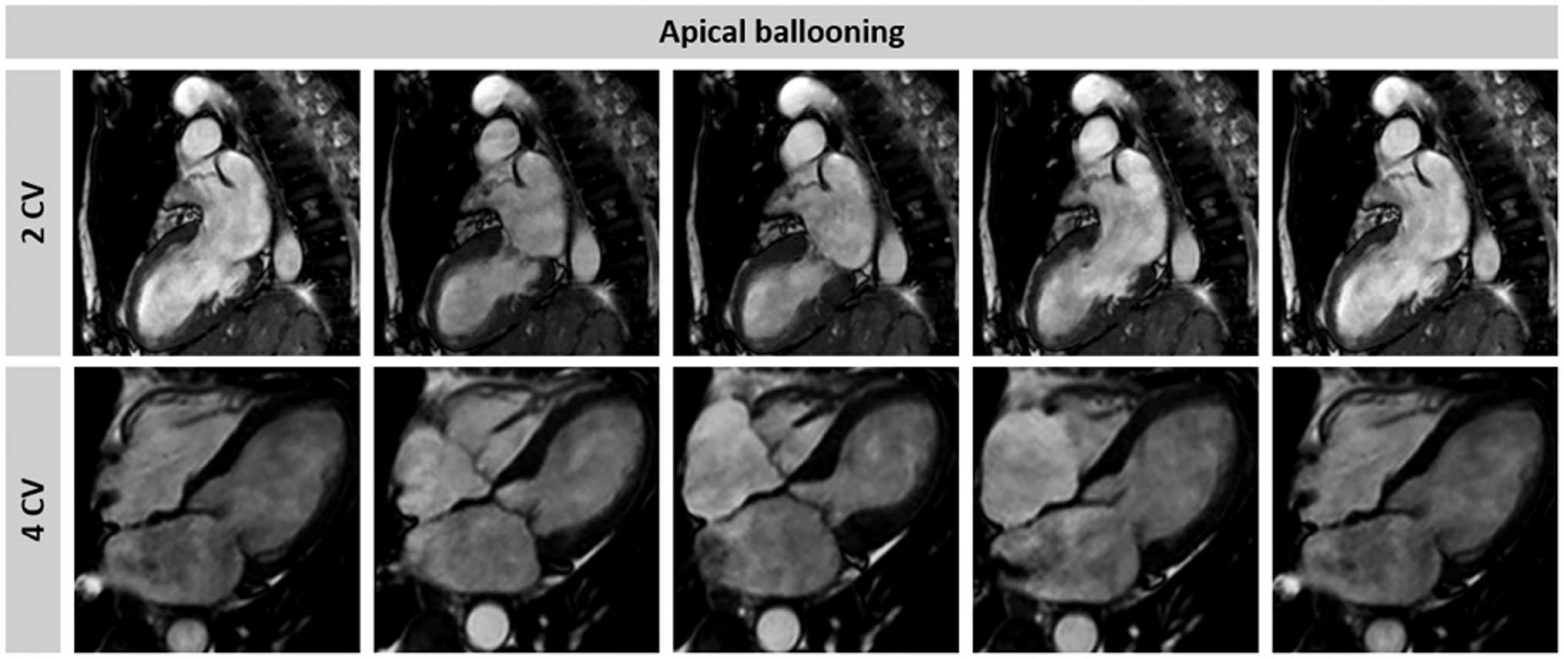
Cine cardiac magnetic resonance of a takotsubo syndrome (TTS) patient. A two- and four-chamber view of the left ventricle in a representative TTS patient with apical ballooning

Balanced steady-state free precession (bSSFP) including short-axis plane covering the entire left ventricle and three long-axis planes (two-chamber, four-chamber, and LVOT view), should be systematically performed during CMR (Supplementary data online, *Videos S7–S10*). These planes enable precise visualization of the contractile dysfunction pattern (Fig. 13) and have an additive role in better defining basal ballooning or focal forms, where limited WMAs can be missed at echocardiography. In addition to a high-resolution illustration of regional LV dysfunction/ballooning, bSSFP images of double oblique short-axis or four-chamber views also enable accurate assessment of RV WMAs. The detection of RV involvement has been associated with prolonged hospital stay and higher rates of short- and long-term adverse events [15, 75–77, 80]. Conversely, due to the complex geometry of the right ventricle, detailed estimation of RV function using TTE can be challenging.

It is also noteworthy that CMR can visualize thrombi in the LV apex, which may cause subsequent systemic embolism, and can detect other complications as well (e.g. pericardial effusion, LVOTO) [96, 97].

**Figure 14:**
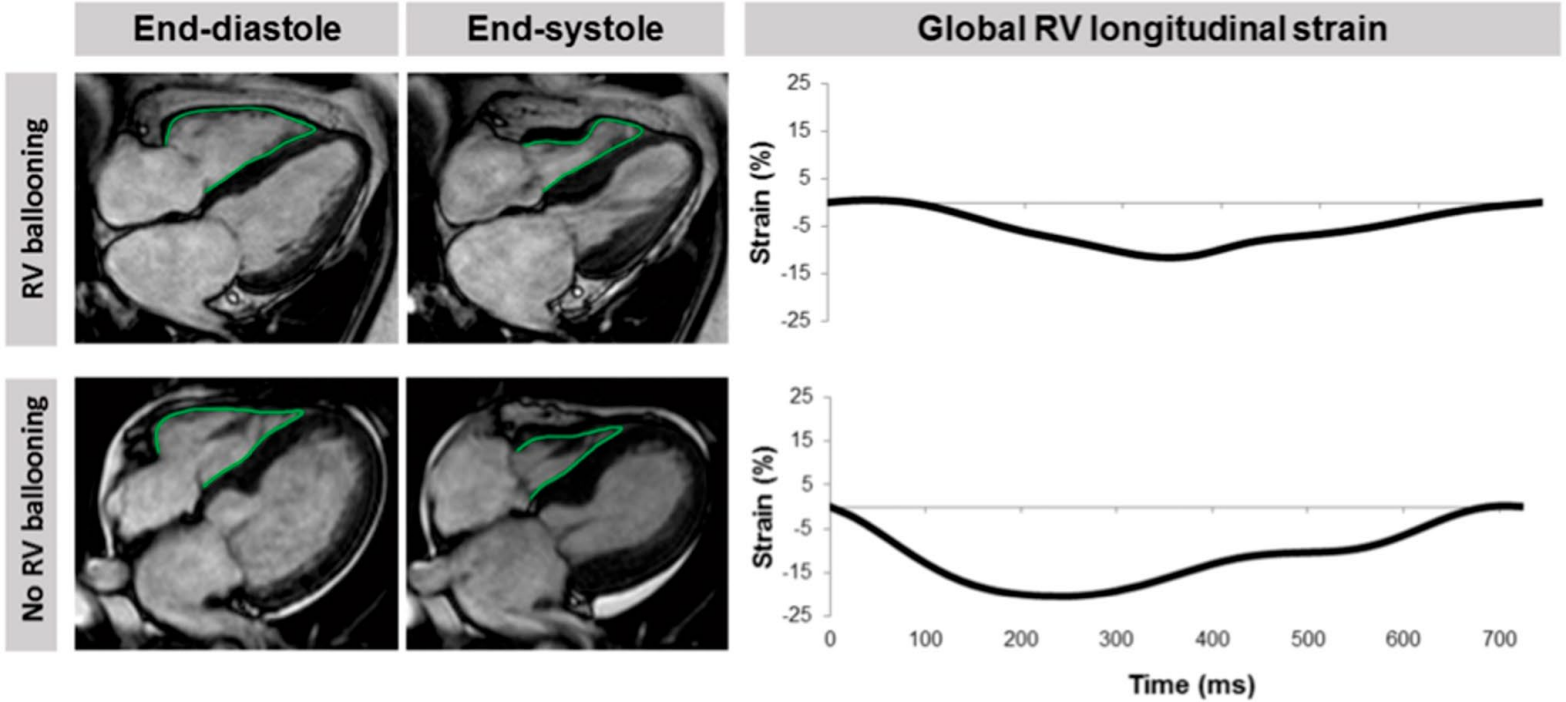
Analysis of right ventricular (RV) longitudinal strain. Cardiac magnetic resonance-feature tracking of long-axis four-chamber views to quantify longitudinal RV strain in a takotsubo syndrome patient with and without RV involvement

Recently, CMR-feature tracking (FT) has been introduced as a new method to quantify regional LV deformation similarly to speckle tracking echocardiography, providing a more reliable and accurate assessment of (dys-)synchrony as well as rotational parameters [98]. In TTS patients, CMR-FT of the left and right ventricles has shown promising results [66, 99]. Transient circumferential dyssynchrony and impaired rotational mechanics are distinct features of TTS with varying degree of severity according to the ballooning pattern. Moreover, the assessment of RV myocardial strain using CMR-FT enables accurate evaluation of RV involvement in TTS and represents a promising approach for optimized risk stratification (Fig. 14). However, the clinical value of these novel parameters for the identification of high-risk patients, as well as the already described transient impairment of left atrial performance, needs to be validated in future prospective studies [100].

### Myocardial tissue characterization

**Figure 15:**
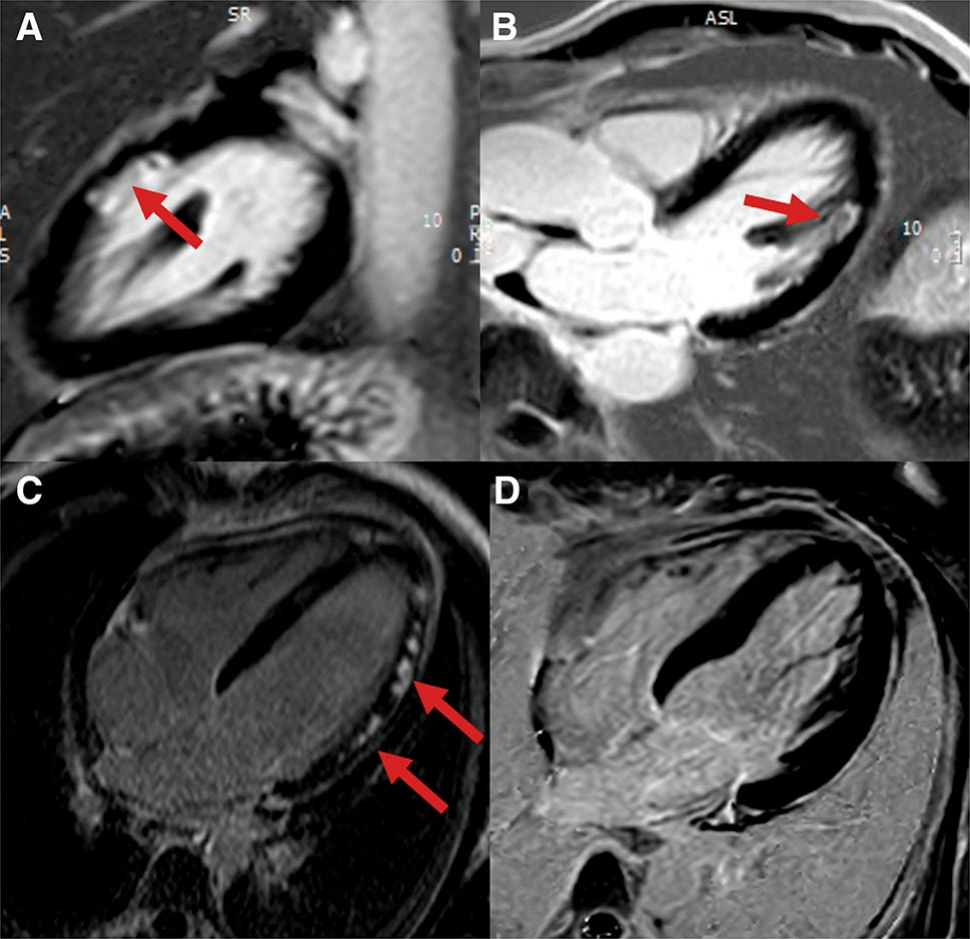
Different late gadolinium enhancement (LGE) patterns in myocardial infarction with non-obstructive coronary arteries (MINOCA). (**a** and **b**) Myocardial infarction with spontaneous lysis of thrombus with subendocardial ischaemic LGE of the lateral wall in two-chamber (**a**) and three-chamber view (**b**). (**c**) Patchy, subepicardial non-ischaemic LGE in a patient with myocarditis (four-chamber view). (**d**) Absence of irreversible tissue injury (LGE) when signal intensity threshold of 5 standard deviation is used in a patient with takotsubo syndrome (four-chamber view)

CMR has the advantage of enabling multiparametric myocardial tissue characterization. Specifically, CMR can rule out ischaemic myocardial injury or myocarditis using late gadolinium-enhancement (LGE) imaging (Fig. 15) [96]. While LGE in myocarditis reveals patchy myocardial necrosis and fibrosis (rarely subepicardial or transmural), the absence of LGE is a quite common finding in patients with TTS and is considered as a diagnostic criterion [101, 102]. However, several studies have reported that, when using LGE signal intensity (SI) threshold of 3 standard deviations (SD), subtle focal or patchy LGE can be detected also in patients with TTS [96, 103–105] Noteworthy, if SI threshold of 5 SDs above the mean remote myocardium is used, no areas of LGE are detectable in TTS patients [96].

Although patchy areas of LGE can be detected in TTS patients using low SI thresholds, the transient nature of this myocardial injury, which generally disappears within few days or weeks, as well as the absence of LGE at high SI thresholds are distinctive patterns that differentiate TTS from AMI and myocarditis. It has been suggested that an increase of extracellular matrix rich in collagen-1, as a finding of transient fibrosis, might be accountable for the LGE observed in TTS [106].

In conclusion, these evidences suggest that the absence of LGE at high SI thresholds is still a diagnostic criterion for TTS, keeping in mind that small amounts of reversible patchy areas of LGE can be transiently detected using low SI thresholds and do not represent an absolute exclusion criterion during diagnostic work-up.

The impact of LGE on clinical outcomes in TTS remains controversial. Although some authors have reported worse clinical prognosis in patients with LGE, no differences in clinical outcomes between patients with and without LGE have been found in a multicentre prospective registry [96, 103].

**Figure 16:**
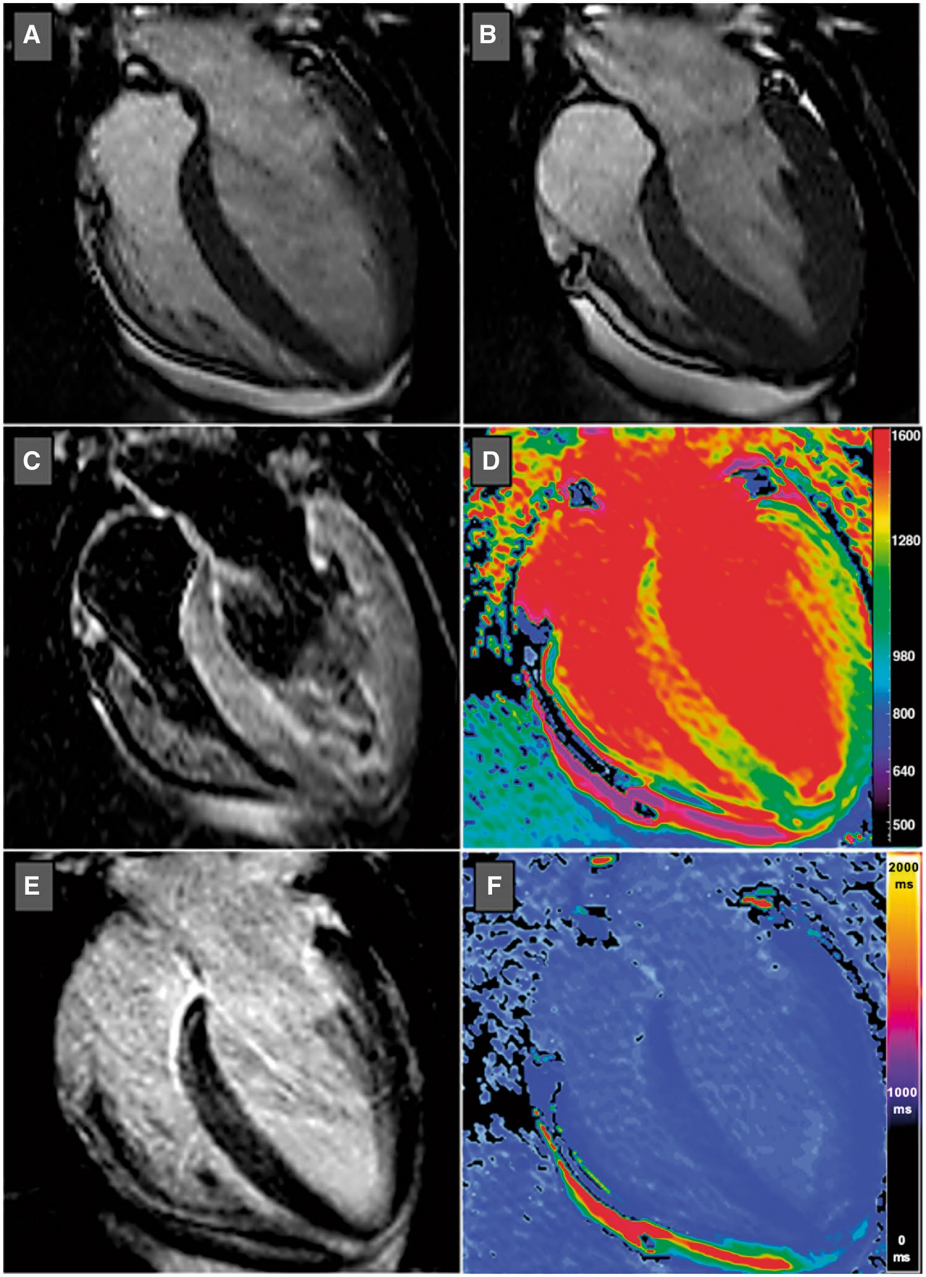
T1 mapping in takotsubo syndrome. End-diastolic (**a**) and end-systolic (**b**) balanced steady-state free precession cardiac magnetic resonance images in horizontal long-axis view showing basal and mid-ventricular hypokinesis and apical hyperkinesis compatible with reversed/basal takotsubo syndrome. Horizontal long-axis views of T2-weighted short tau inversion recovery (**c**) and late gadolinium enhancement (**e**) images revealed subtle hyperintensity and patchy contrast enhancement in the basal and mid-ventricular segments of the left ventricle. Colour-coded native (**d**) and post-contrast (**f**) T1 maps using shortened modified Look-Locker inversion recovery (ShMOLLI) confirmed regional myocardial involvement

T1 mapping techniques, where T1 time of injured myocardium is quantitatively and more objectively assessed, can be useful to detect very subtle areas of damage in patients with TTS (Fig. 16). Assessment by T1 mapping may provide better understanding of TTS pathophysiology, and the first published data have revealed that native T1 values in TTS are significantly higher than in control subjects, suggesting that these new mapping techniques can better identify reversible and irreversible myocardial injury [82].

Up to date, cut-off values of native T1 mapping have not been established in this setting. This technique is still considered a research tool and its impact on clinical practice and outcome needs to be further investigated.

**Figure 17:**
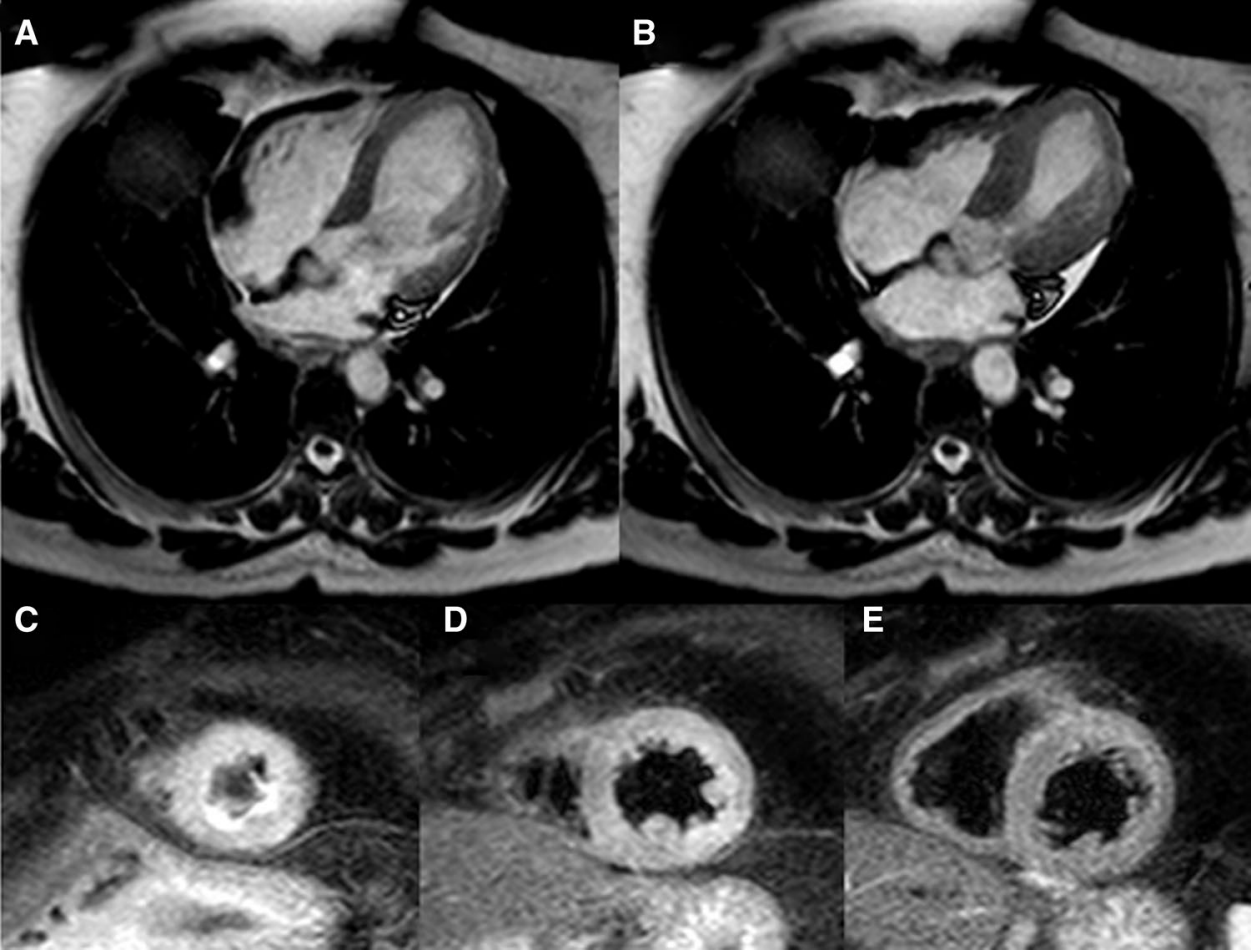
Myocardial oedema in takotsubo syndrome (TTS). TTS patient with typical apical ballooning displayed in end-diastolic (**a**) and end-systolic (**b**) bSSFP imaging in horizontal long-axis view. T2-weighted imaging (short tau inversion recovery) demonstrates circumferential myocardial oedema in the left ventricular apical (**c**) and mid-ventricular (**d**) segments without involvement of the basal segments (**e**). bSSFP, balanced steady-state free precession

Myocardial oedema is another distinctive feature of TTS. The exact pathophysiological mechanisms underlying the development of myocardial oedema in TTS is still unclear. Increased LV wall stress and/or transient ischaemia seem to be responsible for inflammatory injury of cell membrane, with subsequent transmembranous leakage of water and large molecules leading to extracellular oedema. It can be detected by T2 weighted black blood imaging (T2BB) or newer mapping techniques (i.e. native T1 mapping and T2 mapping). T2BB allows to recognize oedema due to the suppression of both fat and flowing blood signal. In T2BB imaging, a triple inversion recovery fast-spin echo sequence is used in contiguous short-axis views of the left ventricle. A ratio of mean SI of the myocardium compared with that of the skeletal muscle (T2 SI ratio) ≥1.9 is generally accepted to define oedema. Myocardial oedema is generally localized in segments with abnormal contraction (a-hypokinesis) showing a diffuse or transmural distribution. Its acute extent also correlates with acute release of both catecholamines and N-terminal pro-B-type natriuretic peptide [82]. It is a transient and reversible phenomenon, typically detectable during the acute phase, which gradually disappears within few weeks along with the recovery of myocardial contractility. Oedema is a key diagnostic feature for assessing *in vivo* the acuity, extent, and severity of myocardial stunning in TTS [108] (Fig. 17).

### When to perform CMR in TTS

In the acute phase, CMR should be used, whenever possible, when echocardiographic images are suboptimal, given its ability to provide more detailed information about ventricular morphology and function, RV involvement, intraventricular thrombi, and pericardial effusion. Moreover, in case of an ambiguous clinical picture, CMR is recommended to detect the presence of oedema, a hallmark finding of TTS, and to rule out other forms of MINOCA in order to adopt the appropriate therapeutic strategy. Being myocardial stunning in TTS transient and reversible, complete, or near complete recovery is expected and necessary to retrospectively confirm TTS diagnosis. In the post-acute phase, CMR is mandatory within 1 month of TTS onset in all patients without an identifiable trigger event (Class III TTS). Additionally, CMR is recommended in case of incomplete recovery (persisting ECG changes and/or WMAs at echocardiography) and atypical presentation (Class II TTS secondary to other illness, e.g. sepsis, pancreatitis, cholecystitis, bone fracture, asthma crisis, cardiac and non-cardiac surgery, and anaesthesia) to assess tissue characterization and definitively corroborate TTS diagnosis.

Key points

- CMR can visualize the entire spectrum of functional and structural changes that occur in patients with TTS.
- CMR diagnostic criteria for TTS include the combination of:
  - typical regional WMAs (apical, mid-ventricular, or basal ballooning);
  - presence of reversible tissue injury (oedema);
  - absence of irreversible tissue injury (LGE) when SI thresholds of 5 standard deviations are used.
    - CMR provides additional value to other imaging modalities for differential diagnosis (myocardial infarction, myocarditis), pathophysiological insights, and detection of complications (e.g. LV thrombi) in TTS.
    - In the acute phase, CMR is recommended in doubtful TTS cases, especially if diagnosis of another type of MINOCA (e.g. myocarditis) requires a different therapeutic approach.
    - In the post-acute phase, CMR is mandatory in all patients within 2 months, especially in case of persisting ECG abnormalities and/or regional WMAs at echocardiography, in order to definitively confirm the diagnosis of TTS.

## Coronary computed tomography angiography

Technological developments over recent years have emphasized the role of coronary computed tomography angiography (CCTA) in the evaluation of patients with suspected TTS [109–111].

An advantage of both CCTA and CMR is the ability to evaluate the heart in any potential plane, overcoming the limitations imposed by suboptimal acoustic windows on echocardiography [112]. Moreover, unlike CA, this imaging modality is not invasive and, compared with CMR, can be readily used in the emergency setting due to its accessibility and fast acquisition time. The main indication for CCTA in TTS patients relates to the assessment of the epicardial coronary arteries and the ruling out of coronary artery disease, according to local expertise and availability [113].

CCTA has been proposed as a non-invasive alternative to CA in stable and pain-free patients with no ST-segment elevation at onset and convincing clinical (post-menopausal woman, trigger event) and echocardiographic (apical ballooning, circumferential pattern, distal LAD flow visualization) findings consistent with TTS. This approach could be advantageous, particularly in patients with delayed presentation (>48 h after chest pain onset) or when CA is not available on site [8, 17].

Cardiac CT, beyond coronary artery disease, allows assessment of LV function and WMAs. Nowadays, cardiac CT also provides myocardial characterization with late iodine enhancement imaging comparable with that obtained from CMR [114].

Owing to multiplanar imaging [115, 116] that overcomes the limitations of echocardiography, multidetector CT permits a better identification of LV apical thrombi, a not so rare complication of TTS. Cardiac CT plays an important role in the diagnostic workup of patients with acute chest pain and doubtful TTS diagnosis to exclude other conditions such as pulmonary embolism and aortic dissection [117, 118]. Recently, a comprehensive assessment of the coronary arteries, LV function, and myocardial late iodine enhancement has been performed in patients with suspected TTS [119], thus pointing to the growing importance of CCTA.

Up to date there is no evidence for delaying invasive CA in patients with ongoing acute chest pain and acute ECG changes. In such a clinical scenario, patients should be managed according to current guidelines for STEMI or ACS until culprit coronary artery disease has been ruled out [20, 21].

CCTA may be chosen instead of CA:

1. in stable patients with low suspicion of ACS;
2. in patients with suspected TTS recurrence;
3. in other conditions generally associated with TTS such as critical illnesses (particularly sepsis, subarachnoid haemorrhage, or ischaemic stroke).

Moreover, CCTA can be considered as a non-invasive valuable alternative in life-threatening conditions where CA is highly likely to cause complications (e.g. terminal malignancy and advanced age with frailty).

Key points

- The main indication for CCTA in TTS patients relates to the assessment of the epicardial coronary arteries and the ruling out of coronary artery disease.
- Cardiac CT also plays an important role in the diagnostic workup of patients with acute chest pain and doubtful TTS diagnosis to exclude other conditions such as pulmonary embolism and aortic dissection.
- CCTA may be chosen instead of CA in some peculiar situations:
  - Stable patients with low suspicion of ACS;
  - Patients with a history of TTS and suspected recurrence;
  - Critical clinical conditions usually associated with TTS (e.g. sepsis, subarachnoid haemorrhage, or ischaemic stroke) or where CA is highly likely to cause complications (e.g. terminal malignancy and advanced age with frailty).

## Nuclear imaging: single-photon emission computed tomography and positron emission tomography

Although the role of nuclear imaging in TTS has not yet been well established in clinical practice, nuclear imaging can provide assessments of myocardial perfusion and metabolic activity, improving our understanding of the pathophysiology of TTS and potentially aiding diagnosis.

**Figure 18:**
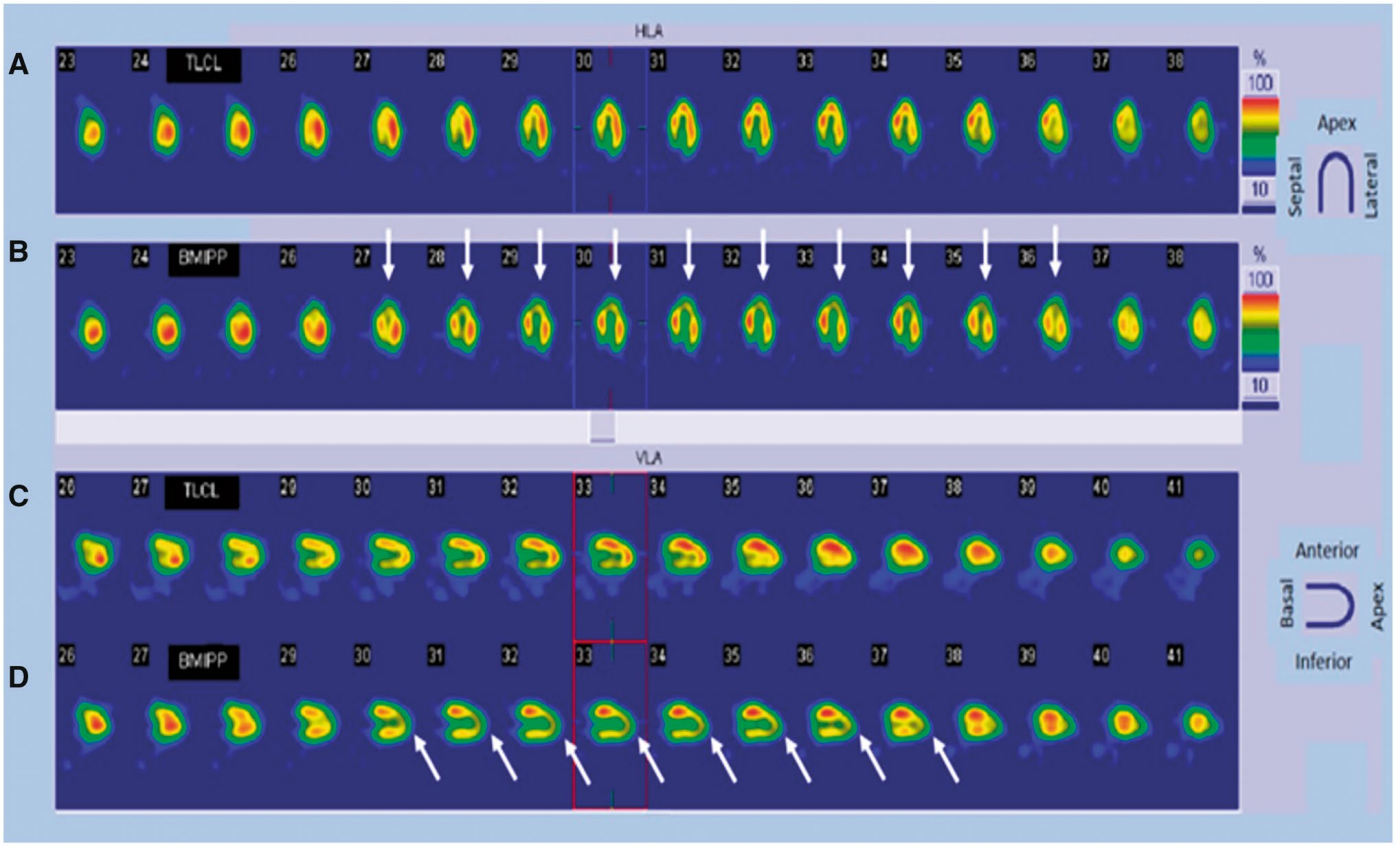
Thallium scintigraphy (**a** and **c**) and cardiac SPECT images using ^123^I-BMIPP (**b** and **d**) in a 76-year-old female patient with takotsubo syndrome. A discrepancy is present between ^123^I-BMIPP and perfusion uptake in the apical area. White arrows show decreased uptake of BMIPP in the apical area, compared with uptake of thallium. ^123^I-BMIPP, ^123^I-beta-methy-iodophenyl pentadecanoic acid; SPECT, single-photon emission computed tomography

Some studies have reported that fatty acid metabolism depicted by ^123^I-beta-methy-iodophenyl pentadecanoic acid (^123^I-BMIPP) single-photon emission computed tomography (SPECT) was more severely impaired than myocardial perfusion depicted by ^201^thallium scintigraphy in acute TTS patients [28, 120]. Reductions in perfusion tracer counts occur as a result of regional myocardial wall thinning at the apex, due to both artefacts and partial volume effects, and may mimic ACS. Combined perfusion and metabolic imaging can help identify this phenomenon and aid the diagnosis of TTS (Fig. 18).

**Figure 19:**
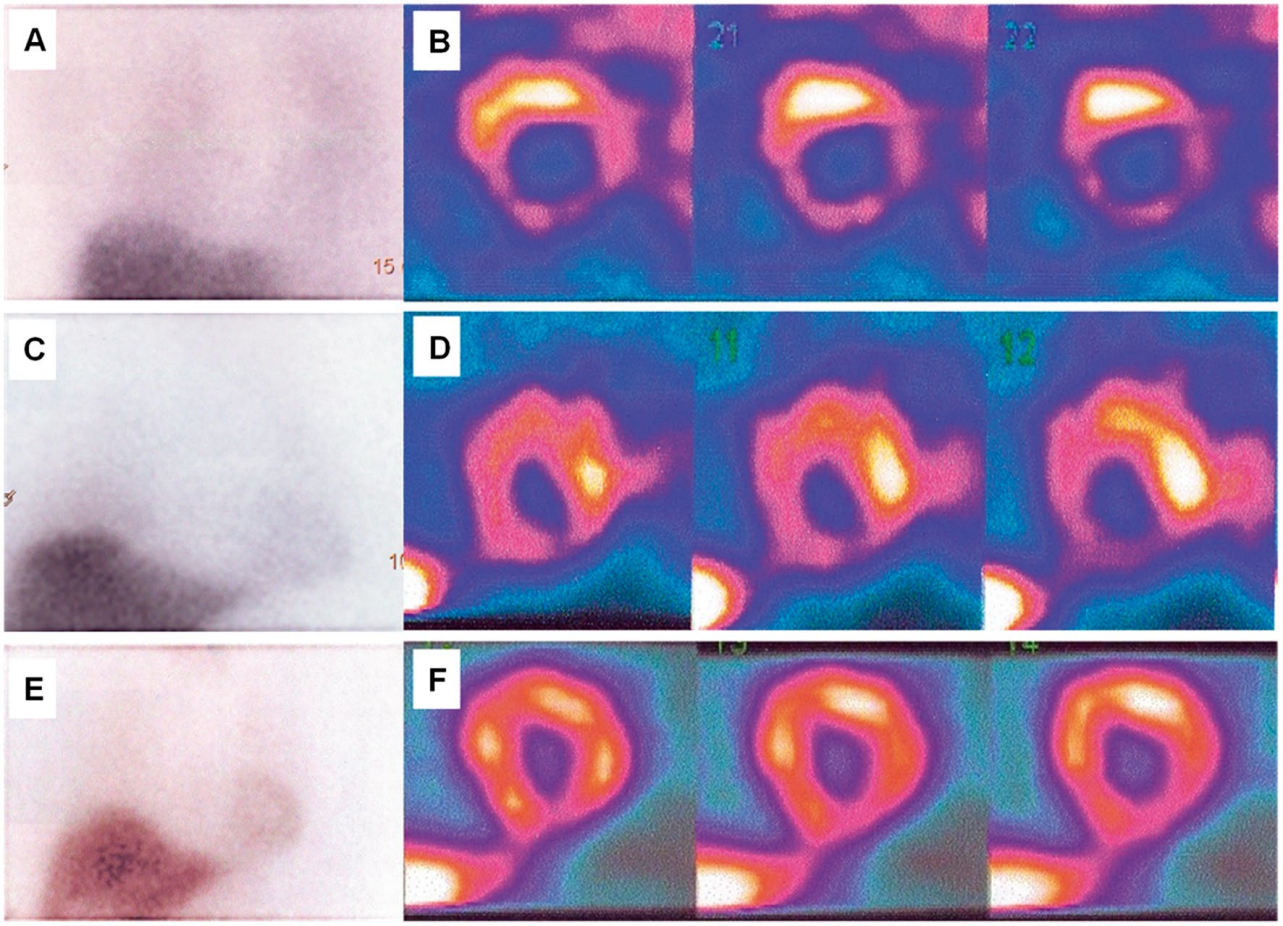
Planar ^123^I-MIBG late images (**a, c, e**) and selected short-axis slices (**b, d, f**) from SPECT late acquisition. (**a, b**) Reduced late heart to mediastinum ratio (1.3) and a clear apical defect on SPECT images. (**c, d**) Mild reduction of the heart to mediastinum ratio (1.5) and a slight apical defect on SPECT images. (**e, f**) Normal heart to mediastinum ratio (1.8) and normal distribution of radiotracer on SPECT images. ^123^I-MIBG, ^123^I-metaiodobenzyl-guanidine; SPECT, single-photon emission computed tomography. (Reprinted with permission from Citro et al. [26])

^123^I metaiodobenzyl guanidine (MIBG) scintigraphy can assess myocardial sympathetic nerve terminal activity and detect adrenal and ectopic pheochromocytoma. Even in the subacute phase, the adrenergic hyperactivity observed in TTS results in decreased uptake and increased washout of ^123^I-MIBG from the heart (Fig. 19) [26, 121]. Indeed, myocardial ^123^I-MIBG uptake is impaired for months as a consequence of regional disturbances in sympathetic neuronal activity [122]. The combination of ^123^I-MIBG and myocardial perfusion scintigraphy is also useful for distinguishing TTS from ACS when innervation and myocardial scarring are matched.

Cardiac positron emission tomography (PET) using [^18^F]2-fluoro-deoxy-glucose (FDG) shows abnormal glucose metabolism alongside normal myocardial perfusion in TTS. Recently, some studies have been conducted at different phases during the TTS time course [123, 124]. In the acute and subacute phases, similar defects of MIBG and FDG uptake were found, despite slightly reduced perfusion in TTS. Although rapid normalization of myocardial perfusion was observed, TTS patients had delayed recovery of both LV glucose metabolism and sympathetic innervation. In the context of normalized LV wall motion, the delayed recovery of glucose metabolism and sympathetic innervation may allow diagnosis in patients with delayed presentation, even within a few months of the suspected triggering episode.

The pathophysiology of TTS remains challenging and controversial. Several hypotheses have been proposed to explain this unique cardiac disorder [52, 125–128], which can be broadly categorized as vascular or myocardial causes. Temporal evolution of MIBG and FDG abnormalities has provided new insight into functional myocardial changes and their time course in TTS; however, the mechanisms of TTS remain undetermined. The diagnostic criteria of TTS have been updated, such that coronary artery disease and pheochromocytoma are included in the new criteria. Therefore, nuclear imaging seems to be an ever more important imaging modality for the assessment of TTS, not only for diagnostic purposes, but also as a research tool that can allow better understanding of the pathophysiology of TTS.

Key points

- Although the role of nuclear imaging in TTS has not yet been well established in clinical practice, myocardial perfusion and sympathetic nerve innervation can be assessed by SPECT and MIBG.
- In the context of normalized LV wall motion, the delayed recovery of glucose metabolism (by FDG-PET) and sympathetic innervation (by ^123^I-MIBG scintigraphy) may allow diagnosis of TTS in patients with delayed presentation, even within a few months of the suspected triggering episode.

## Why and how to recognize TTS in COVID-19 era

Coronavirus disease 2019 (COVID-19) is a tremendous infectious disease firstly recognized in Wuhan, Hubei, China, that has spread throughout to other provinces in China and impetuously involved worldwide many countries causing a pandemic and a public health crisis of global proportions in 2020. Cardiovascular involvement, including acute coronary syndromes, myopericarditis, and pulmonary embolism, has been associated to the clinical course of a substantial proportion of patients affected by severe acute respiratory syndrome coronavirus 2 (SARS-CoV-2). Myocardial injury emerged as an important predictor of worst outcome. Shi et al. report a prevalence of 19.7% of cardiac injury, defined by high blood levels of high-sensitivity troponin I, among 416 patients with confirmed diagnosis of SARS-CoV-2 [129]. In this study, cardiac injury was associated with higher risk of in-hospital mortality. Their results are consistent with another study by Guo et al. on 187 Chinese patients, which highlighted the association between myocardial injury and fatal outcome in SARS-CoV-2 patients [130].

As with other coronaviruses, an intense systemic inflammatory response secondary to SARS-CoV-2 may produce demand ischaemia and/or possible coronary plaque disruption. Moreover, the detection of increased levels of multiple cytokines and chemokines in patients hospitalized in intensive care unit, suggests a direct myocardial damage as an alternative potential mechanism [131].

Noteworthy septic status, hypoxaemia, and metabolic acidosis; complications often detected in SARS-CoV-2 patients, are established triggers for secondary TTS.

Owing to the detection of myocardial inflammatory infiltrates documented in TTS patients, possible shared pathophysiological pathways in occurrence of myocardial dysfunction might be hypothesized in patients with SARS-CoV2 experiencing myocardial injury [82]. TTS and its possible complications such as acute heart failure, cardiogenic shock and life-threatening arrhythmias should be taken into consideration in differential diagnosis among various cardiovascular presentations associated with SARS-CoV-2 [132].

Imaging diagnostic tests are not routinely used in the emergency context of pandemic disease, the European Association of Cardiovascular Imaging recommend performing echocardiography at least in patients with laboratory or electrocardiographic signs of cardiac injury [133]. The suspect of TTS should arise in patients with echocardiographic evidence of extensive myocardial dysfunction especially if typical apical ballooning pattern is detected [134]. Clinicians should consider combining coronary CT with lung scanning (usually used for pneumonia disease) to exclude coronary artery disease. Multislice computed tomography (MSCT), being able to detect late iodine enhancement and myocardial fibrosis, is an additional tool to exclude myocarditis in this clinical context. Compared with MSCT, CMR is able to recognize myocardial oedema, a hallmark sign of TTS playing a key role in differential diagnostic work-up [135].

Although the current challenging clinical scenario makes serial invasive and not invasive diagnostic testing difficult, whenever possible multimodality imaging should be performed in patients with SARS-CoV-2 and cardiac injury to distinguish type II myocardial infarction, myocarditis, and TTS, and to adopt the most appropriate therapeutic strategy.

## Diagnostic algorithm and differential diagnosis

**Figure 20:**
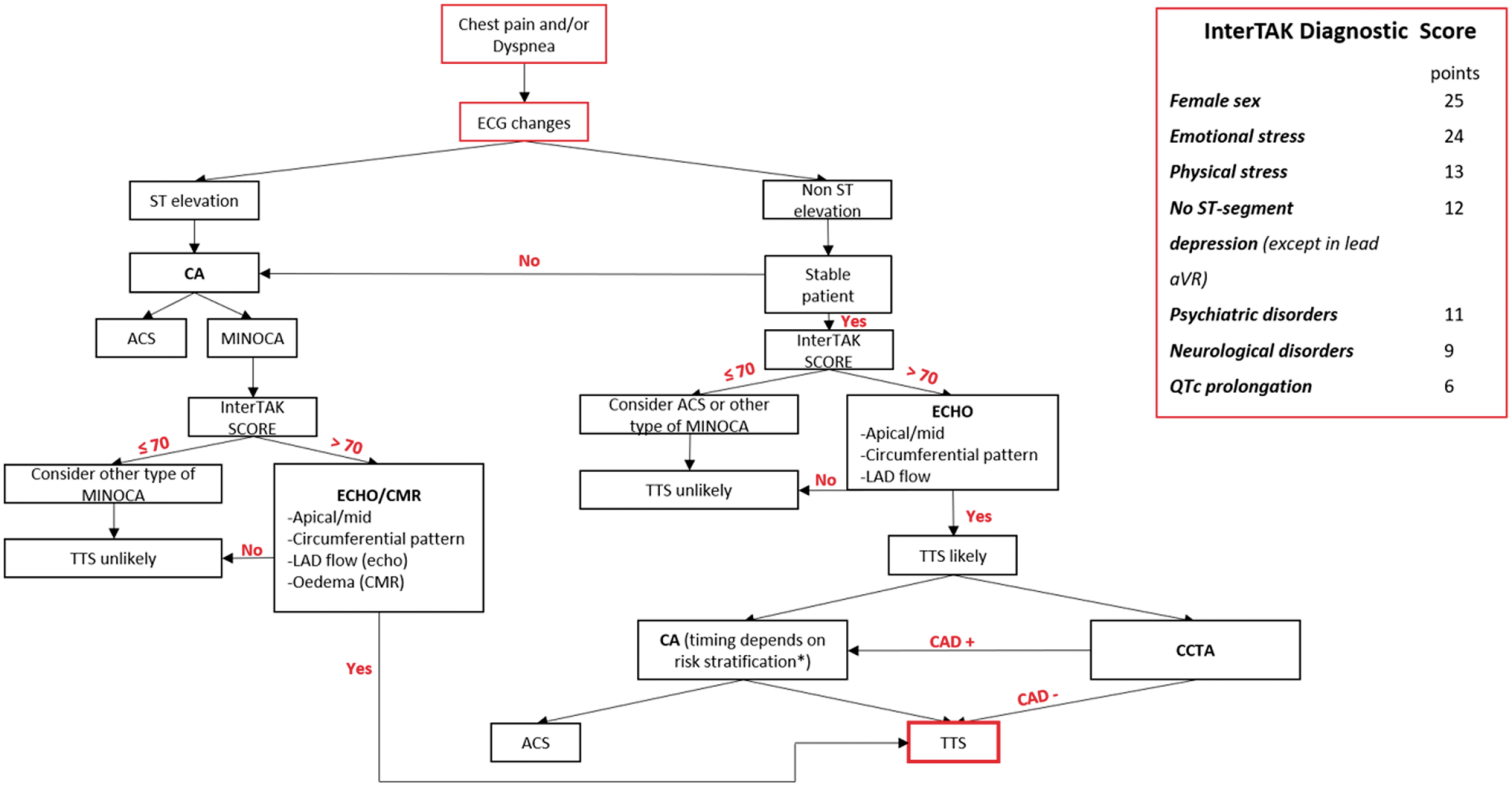
Diagnostic algorithm in patients with clinical suspicion of takotsubo syndrome at presentation. *Recommendations for invasive coronary angiography and revascularization in non-ST-elevation acute coronary syndromes should be followed. ACS, acute coronary syndrome; CA, coronary angiography; CAD, coronary artery disease; CCTA, coronary computed tomography angiography; CMR, cardiac magnetic resonance; ECG, electrocardiogram; LAD, left anterior descending artery

**Figure 21:**
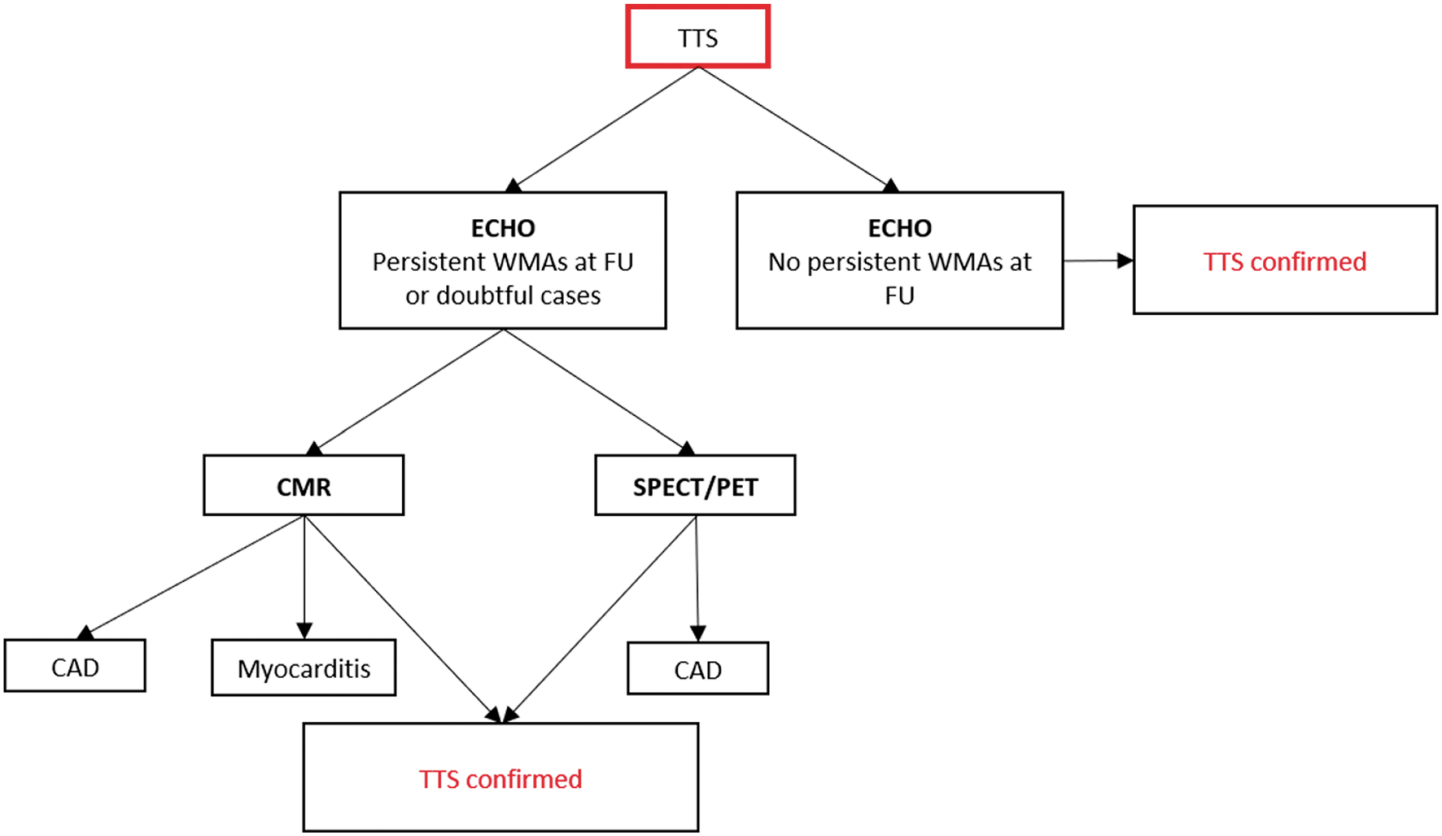
Role of multimodality imaging during post-acute phase in patients with diagnosis of takotsubo syndrome. CAD, coronary artery disease; CMR, cardiac magnetic resonance; FU, follow-up; PET, positron emission tomography; SPECT, single-photon emission computed tomography; TTS, takotsubo syndrome; WMAs, wall motion abnormalities

TTS is a complex clinical entity, with a wide spectrum of clinical presentations and varying levels of risk and haemodynamic stability, which requires tailored management strategies depending on the different scenarios. Multimodality imaging plays a key role in establishing the diagnosis, guiding therapy and stratifying prognosis in both the acute and post-acute phases of TTS (Figs. 20 and 21; Table 5). In clinical practice, echocardiography is the first-line imaging tool due to its widespread availability and feasibility in the acute care setting and the ability to easily and rapidly monitor changes in cardiac function over time. In asymptomatic or paucisymptomatic patients, the detection of unexplained extended apical WMAs, despite mild ST-T changes at ECG and low troponin elevation, is highly suggestive of TTS diagnosis, especially if predisposing factors exist. Patients presenting with acute chest pain and/or dyspnoea, after ECG and first clinical evaluation, can be screened by the InterTAK score. If the score is >70, echocardiographic findings compatible with apical or mid-ventricular ballooning, circumferential pattern and antegrade LAD flow, will orient the diagnosis towards TTS. In this case, coronary anatomy may be assessed invasively by CA or non-invasively by CCTA. Once CCTA and/or CA have confirmed the absence of culprit atherosclerotic plaques, the patient should be hospitalized and monitored in the intensive care unit for at least 48–72 h. TTE should be performed earlier in order to assess EF, cardiac output and, especially in unstable patients, to detect the onset of functional and mechanical complications (i.e. LVOTO, severe MR, and RV involvement). FU echocardiography is recommended daily or every two or three days during the first weeks, and at longer intervals after the acute phase. TTE allows monitoring of the recovery of transient WMAs and can stratify patients into ‘low risk’ and ‘high risk’, the latter requiring specific therapeutic measures. Being able to provide more detailed information about LV morphology and function, RV involvement, intraventricular thrombi, and pericardial effusion, CMR can be useful in the acute phase but its use is conditioned by the more limited availability, compared with echocardiography, and the difficulties in imaging unstable patients. Conversely, owing to its ability to detect the presence of oedema, a hallmark finding in TTS diagnosis, CMR is crucial during the post-acute phase to rule out other common pathologies (e.g. ACS and myocarditis) (Table 6). It should be performed in doubtful cases or in patients with persistent WMAs, even after discharge. Of note, recent studies have demonstrated that long-term FU in TTS patients is not uneventful. Further studies are needed to verify if assessment of subclinical myocardial dysfunction by speckle tracking echocardiography and other more sophisticated nuclear techniques (e.g. SPECT-MIBG, PET) can help identify patients that may be more prone to develop recurrences, life-threatening arrhythmias, or heart failure phenotype.

**Table 5:** Strengths and weaknesses of non-invasive multimodality imaging in takotsubo syndrome

	Echocardiography	CMR	CTA	Nuclear imaging
Accessibility	++++	++	+++	++
Cost	+	+++	++	++
Radiation risk	–	–	++	++++
LV morphology and function	+++	++++	++	+++
RV function	++	++++	++	–
MR quantification	+++	+++	–	–
LVOTO	++++	++	–	–
LV/RV thromb	++	++++	+++	–
Tissue characterization	+	++++	++	+
Coronary artery imaging	+	++	++++	–
Differential diagnosis ^a^:				
CAD	++	++++	++++	+++
MINOCA	+	++++	+++	++
Myocarditis	++	++++	++	+
Usefulness in FU	+++	+++	–	++

^a^CTA can be useful to exclude pulmonary embolism and aortic dissection.

+, low; ++, medium; +++, high; ++++, excellent; CAD, coronary artery disease; CMR, cardiac magnetic resonance; CTA, computed tomography angiography; FU, follow-up; LV, left ventricular; LVOTO, left ventricular outflow tract obstruction; MINOCA, myocardial infarction with non-obstructive coronary arteries; MR, mitral regurgitation; RV, right ventricular; – = none.

**Table 6:**
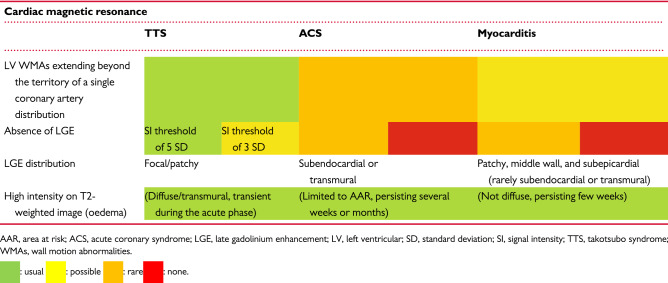
Differential diagnosis of takotsubo syndrome with cardiac magnetic resonance

## Electronic supplementary material

Below is the link to the electronic supplementary material.

Supplementary file1 (AVI 2875 kb)

Supplementary file2 (AVI 2992 kb)

Supplementary file3 (AVI 11181 kb)

Supplementary file4 (MP4 1033 kb)

Supplementary file5 (MP4 1028 kb)

Supplementary file6 (MP4 1231 kb)

Supplementary file7 (AVI 2389 kb)

Supplementary file8 (AVI 18591 kb)

Supplementary file9 (AVI 2065 kb)

Supplementary file10 (AVI 2053 kb)
